# In Silico Prediction of Molecular Targets of Astragaloside IV for Alleviation of COVID-19 Hyperinflammation by Systems Network Pharmacology and Bioinformatic Gene Expression Analysis

**DOI:** 10.3389/fphar.2020.556984

**Published:** 2020-09-16

**Authors:** Chenliang Ge, Yan He

**Affiliations:** ^1^ Department of Geriatrics Cardiology, The First Affiliated Hospital of Guangxi Medical University, Nanning, China; ^2^ Department of Cardiology, The First Affiliated Hospital of University of South China, Hengyang, China

**Keywords:** COVID-19, Astragaloside IV, hyperinflammation, network pharmacological, cytokine storms****

## Abstract

**Introduction:**

The overproduction of cytokines and chemokines caused by excessive and uncontrolled inflammation contributes to the development of COVID-19. Astragaloside IV is considered as an anti-inflammatory and antioxidant agent. This study aimed at undertaking a network pharmacology approach and bioinformatics analysis to uncover the pharmacological mechanisms of Astragaloside IV on COVID-19.

**Methods:**

Potential targets of Astragaloside IV were screened from public databases. Differentially expressed genes (DEGs) in SARS-CoV-2 were screened using bioinformatics analysis on the Gene Expression Omnibus (GEO) datasets GSE147507. Gene ontology (GO) and Kyoto Encyclopedia of Genes and Genomes (KEGG) pathway enrichment analyses were subsequently performed. The overlapping genes, GO terms and KEGG pathways between Astragaloside IV targets and SARS-CoV-2 DEGs were confirmed, and the location of overlapping targets in the key pathways was queried using KEGG Mapper.

**Results:**

A total of 425 potential targets of Astragaloside IV were screened. Besides, a total of 546 DEGs were identified between SARS-CoV-2 infected samples and control samples, including 380 up-regulated and 166 down-regulated genes. There was a significant overlap in GO terms and KEGG pathways between Astragaloside IV targets and SARS-CoV-2 DEGs. The shared genes included MMP13, NLRP3, TRIM21, GBP1, ADORA2A, PTAFR, TNF, MLNR, IL1B, NFKBIA, ADRB2, and IL6.

**Conclusions:**

This study is the first to propose Astragaloside IV as a new drug candidate for alleviating hyper-inflammation in COVID-19 patients. Besides, the key targets and pathways may reveal the main pharmacological mechanism of Astragaloside IV in the treatment of COVID-19.

## Introduction

COVID-19 has reached pandemic proportions around the world. Severe acute respiratory distress syndrome (ARDS) represents an important clinical feature of COVID-19, and a primary cause of death in COVID-19 patients (McGonagle et al., 2020; Ramanathan et al., 2020; Wu et al., 2020). In response to SARS-CoV-2 infection, immune cells and nonimmune cells release large amounts of proinflammatory cytokines, which lead to “cytokine storms”. Clinical studies have revealed that COVID-19 patients admitted to intensive care have increased expression of inflammatory cytokines (IL-6, IL-10, IL-2, and IFN-γ). Other studies confirm that a large number of patients with severe COVID-19 are likely to suffer a cytokine storm syndrome (Huang et al., 2020; Shen et al., 2020; Ye et al., 2020). ARDS and cytokine storms occur very often in patients with COVID-19 since excess production of pro-inflammatory cytokines results in ARDS aggravation (Howell and Davis, 2018). Therefore, there a need to search for therapies to reduce hyper-inflammation and improve prognosis in severe COVID-19 patients.

Huangqi (Radix Astragali Mongolici) is a Well-Known Chinese Tonic. According to the Chinese Pharmacopeia, it is the dried root of the leguminous plants Astragalus mongholicus. It has been widely used in ischemic cardio-cerebrovascular disease, viral hepatitis, kidney disease, and skin diseases for the nourishment of Qi and blood (Li et al., 2017; Gong et al., 2018).

More than 40 active constituents of astragalus root have been identified, with Astragaloside IV (PubChem, CID13943297) being the major active compound.

The pharmacopeia of the People’s Republic of China has regarded Astragaloside IV as the quality standard for Astragalus membranaceus injection (Cao et al., 2019; Wang C. J. et al., 2020). Astragaloside IV has been widely used due to its anti-inflammatory effects associated with various molecular mechanisms (Ren et al., 2013; Li et al., 2017). Astragaloside IV has been reported to decrease *TNF-α, IL-1β* release, and the expression of other inflammatory cytokines by inhibiting the phosphorylation of *IκB* and decrease nuclear translocation of *NF-κB* (Huang et al., 2017). Astragaloside IV also suppresses neutrophils adhesion-related molecules (Shen et al., 1998; Jin et al., 2010).

**Figure 1:**
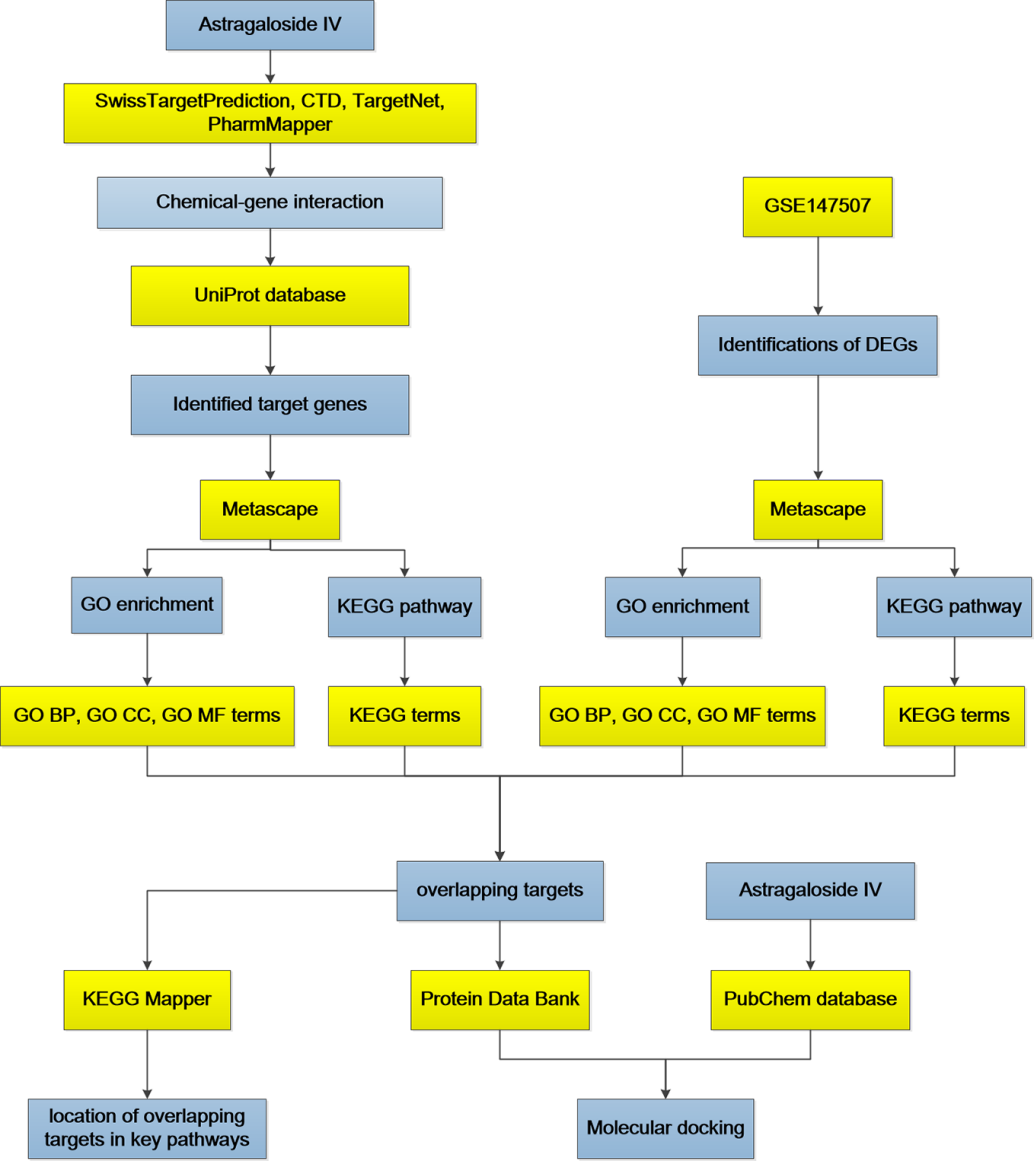
The schematic diagram based on pharmacology analysis.

Currently, no studies have reported the use of Astragaloside IV in the treatment of COVID-19. In this study, the network pharmacology approach and bioinformatics analysis were used to investigate the possible mechanism of action underlying the effectiveness of Astragaloside IV in the treatment of COVID-19. The analysis workflow of network pharmacology is shown in **Figure 1**.

## Materials and Methods

### Potential Astragaloside IV-Related Targets

The 2D molecular structure and PubChem CID of Astragaloside IV were obtained from PubChem (https://pubchem.ncbi.nlm.nih.gov/), which is the world’s largest database of free access to chemical information (Wang et al., 2017). To predict the potential targets for Astragaloside IV with the AUC >0.7, SwissTargetPrediction(http://www.swisstargetprediction.ch/), (Daina et al., 2019), Comparative Toxicogenomics Database (CTD, http://ctdbase.org/about/), (Davis et al., 2019) and TargetNet (http://targetnet.scbdd.com) (Yao et al., 2016) were used. Generate Conformers was confirmed, maximum generated conformations are 300, select targets set of Pharmacophore Mapping was choosed as Druggable Pharmacophore Models, then Astragaloside IV related targets were predicted using PharmMapper (http://www.lilab-ecust.cn/pharmmapper/)(Wang et al., 2017). However, due to the nonstandard naming, the names of the targets were converted to official symbols using the UniProt Knowledgebase (UniProtKB, http://www.uniprot.org/) and species was restricted to “Homo sapiens”.

### SARS-CoV-2 Related Genes

GSE147507 was downloaded from the GEO (http://www.ncbi.nlm.nih.gov/geo/)(Barrett et al., 2013) database. In the publisher’s study, the transcriptional responses of hosts to SARS-CoV-2 and other respiratory infections were systematically described. These data suggest that the unique transcriptional characteristics may be related to the pathogenesis of COVID-19 (Blanco-Melo et al., 2020). The transcriptional results of A549 cells (Series7) were selected for analysis. Impute and limma packages in R provided by the Bioconductor project (http://www.bioconduct-or.org/packages/release/bioc/html/affy.html) (Chen et al., 2007) were used to assess the transcriptional results. Quantile normalization and log2-transformation were performed before analyzing the matrix data. Original p-values were adjusted using the Benjamini-Hochberg method, and fold-changes (FC) were calculated using the false discovery rate (FDR). The |log2 FC|>2 and adj.P.Val <0.05 were used to filter the differentially expressed genes (DEGs).

### KEGG Pathway and GO Enrichment Analysis

A list of SARS-CoV-2 DEGs and Astragaloside IV related targets were submitted to Metascape (http://metascape.org) (Zhou et al., 2019), with the species limited to “Homo sapiens”. Functional enrichment analysis was performed based on the three categories of GO terms; Biological Processes, Cellular Components, and Molecular Functions. All genes present in the GSE147507 dataset were used as the enrichment background. Terms with a p-value < 0.01, a minimum count of 3, and an enrichment factor >1.5 were collected and grouped into clusters based on their membership similarities. The intersection of KEGG Pathways and GO terms were identified between Astragaloside IV-related targets and SARS-CoV-2 DEGs.

### PPI Network Construction and Key Pathways

SARS-CoV-2 DEGs utilized as hub proteins and submitted to String (https://string-db.org/) (Szklarczyk et al., 2019), with species limited to “Homo sapiens”. A confidence score>0.9 was used to obtain the PPI networks, which were visualized using Cytoscape 3.7.2 (http://www.cytoscape.org/) (Otasek et al., 2019). Overlapping targets were identified by considering the intersection of Astragaloside IV-related targets and SARS-CoV-2 DEGs. The location of overlapping targets in critical pathways was queried using KEGG Mapper.

### *In Silico* Molecular Docking Study of Astragaloside IV Key Targets

Autodock Vina was used to carrying out molecular docking of Astragaloside IV key targets (Trott and Olson, 2010). The PDB format molecular structure of Astragaloside IV was obtained from the PubChem database (https://www.ncbi.nlm.nih.gov/). The structures of the key targets were downloaded from the Protein Data Bank (http://www.rcsb.org/). The water molecules were deleted from the protein crystal structure, hydrogen atoms added, and charge calculated. The molecular docking mode of Astragaloside IV to the protein targets was selected as Local Search Parameters. The docking score was used to evaluate the theoretical binding affinities of Astragaloside IV to the key targets. In order to compare the binding affinities of standard pharmacological immunosuppressive drugs with Astragaloside IV, the same methods were used to evaluate binding affinities of Rapamycin to the key targets.

## Results

### Potential Targets of Astragaloside IV

**Figure 2:**
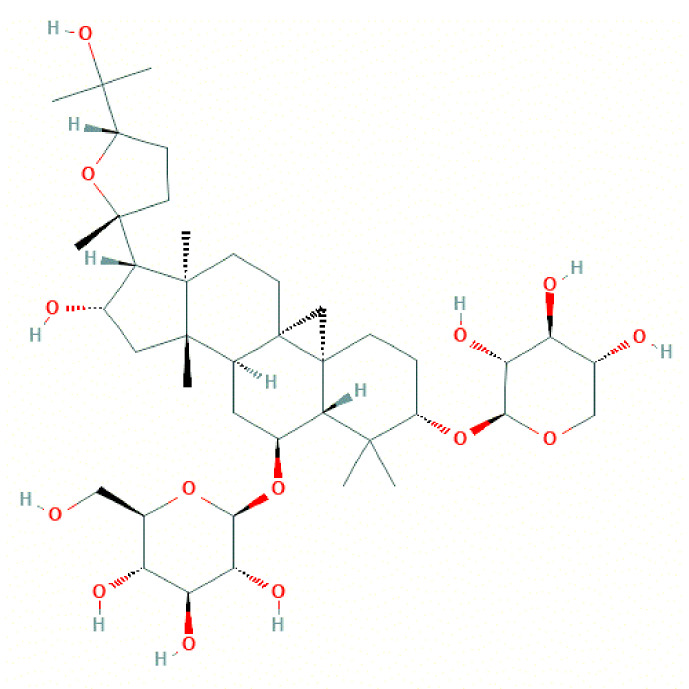
2D molecular structure of Astragaloside IV.

**Figure 3:**
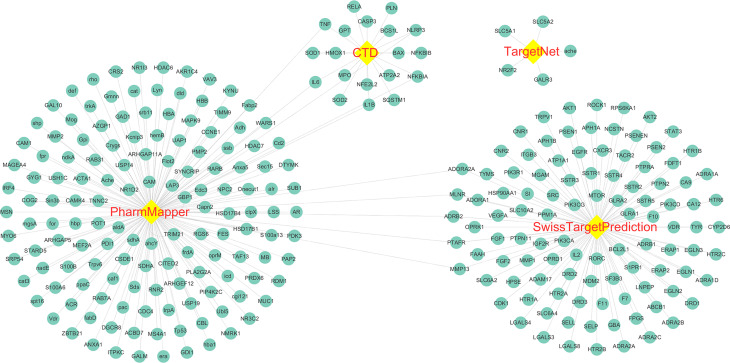
Astragaloside IV related targets. The targets were identified from PharmMapper, Comparative Toxicogenomics Database (CTD), TargetNet, and SwissTargetPrediction.

The molecular structure of Astragaloside IV was download from PubChem (**Figure 2**), and PubChem CID of Astragaloside IV is 13943297. A total of five corresponding potential targets of Astragaloside IV were identified from TargetNet.19, while the corresponding potential targets of Astragaloside IV were identified from CTD. Besides, 154 corresponding potential targets of Astragaloside IV were identified using PharmMapper and 104 corresponding potential targets of Astragaloside IV were identified from SwissTargetPrediction. After data de‐duplication, 282 potential targets were retained (**Figure 3**, **Supplemental file Table S1**).

### Identification of SARS-CoV-2 DEGs

**Figure 4:**
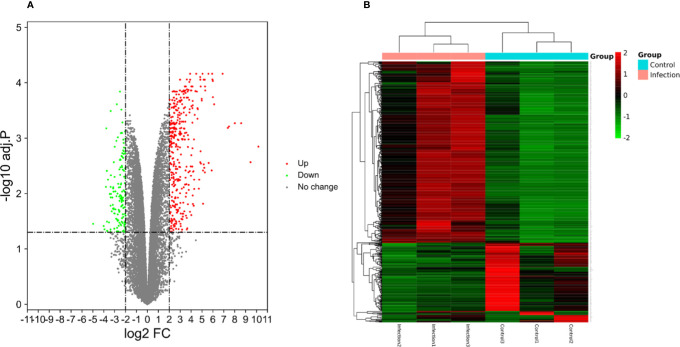
**(A)** The volcano plots displaying significantly expressed (|log2 FC|>2, adj.P.Val <0.05) mRNAs between the control and SARS-CoV-2 groups. Red dots represent up-regulated genes, green dots represent down-regulated genes. **(B)** Heatmaps depicting the expression levels of SARS-CoV-2 differentially expressed genes (DEGs). The upregulated genes are marked in red, the downregulated genes are marked in green.

A total of 23,710 genes and 546 DEGs were identified from SARS-CoV-2 infected samples compared with the control samples, including 380 up-regulated and 166 down-regulated genes. The identified DEGs between the control and SARS-CoV-2 groups were presented in volcano plots. **Figure 4** shows the heatmaps showing genes expression of the DEGs (**Supplemental file Table S2**).

### Gene Ontology and KEGG Enrichment Analysis of Astragaloside IV-Related Targets and SARS-CoV-2 DEGs

**Figure 5:**
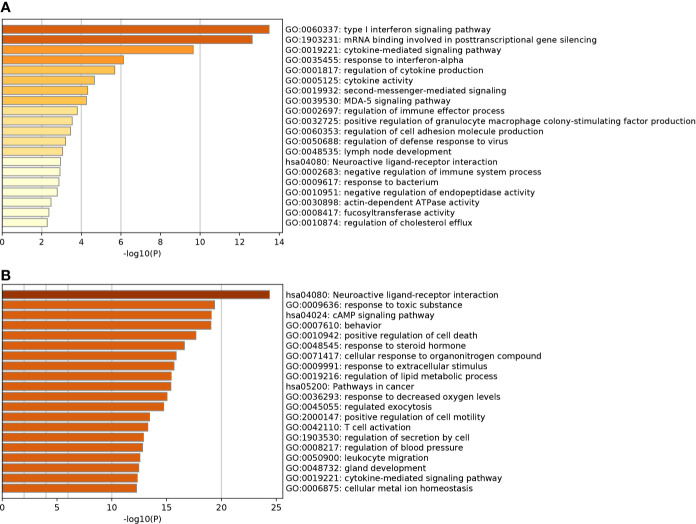
**(A)** Top 20 most significant gene ontology (GO) terms and Kyoto Encyclopedia of Genes and Genomes (KEGG) enrichment analysis for SARS-CoV-2 DEGs. **(B)** Top 20 most significantly GO terms and KEGG enrichment analysis for Astragaloside IV related targets.

Based on GO enrichment, the SARS-CoV-2 DEGs were enriched in 150 terms, 127 in the category Biological Processes, and 23 in Molecular Functions. GO enrichment revealed that the DEGs were mainly involved in various biological processes (BP), including type I interferon signaling pathway, cellular response to type I interferon, response to type I interferon, defense response to a virus, mRNA binding involved in posttranscriptional gene silencing, response to a virus, defense response to other organism and negative regulation of viral genome replication. KEGG analysis revealed that the DEGs were enriched in nine pathways including, Influenza A, Measles, NOD-like receptor signaling pathway, Hepatitis C, Herpes simplex infection, Cytokine-cytokine receptor interaction, Chemokine signaling pathway. Top 20 terms in which the DEGs were enriched and ordered by *p-*value are listed in **Figure 5A**.

The Astragaloside IV related targets were enriched in 385 terms, including 341 in Biological Processes, 13 in Molecular Functions, and 31 in Cellular Components. The targets were involved in biological processes (BP) including, blood circulation, circulatory system process, G protein-coupled receptor signaling pathway, coupled to cyclic nucleotide second messenger, response to toxic substances, the vascular process in the circulatory system, regulation of system process, positive regulation of cell death, and regulation of tube diameter. KEGG analysis revealed that the targets were enriched in 74 pathways, all targets were primarily enriched in neuroactive ligand-receptor interaction, calcium signaling pathway, cAMP signaling pathway, pathways in cancer, and neurotrophin signaling pathway. The top 20 terms in which the targets were enriched and ordered by *p*-value are listed in **Figure 5B**.

The results revealed that there was a significant overlap in GO terms between Astragaloside IV targets and SARS-CoV-2 DEGs. The intersection of Go terms between Astragaloside IV related targets and SARS-CoV-2 DEGs included 34 terms (**Figure 7**). The top 10 overlapping GO terms ordered by *p*-value are listed in **Table 1**.

**Table 1:** Top 10 overlapping gene ontology (GO) terms between Astragaloside IV targets and SARS-CoV-2 differentially expressed genes (DEGs).

Category term	Description	Count	P value
**GO:0002526**	cytokine-mediated signaling pathway	53	2.22498E-10
**GO:0032755**	regulation of cytokine production	41	2.05545E-06
**GO:0010874**	second-messenger-mediated signaling	26	4.79803E-05
**GO:0002438**	positive regulation of cytokine production	27	8.37368E-05
**GO:0002526**	cytokine secretion	17	0.000161558
**GO:0032755**	regulation of cytokine secretion	15	0.000335339
**GO:0002438**	negative regulation of protein secretion	11	0.000653223
**GO:0002438**	negative regulation of peptide secretion	11	0.00095377
**GO:0001730**	cellular calcium ion homeostasis	24	0.001033831
**GO:0002438**	negative regulation of immune system process	24	0.001200594

### PPI Network Analysis

**Figure 6:**
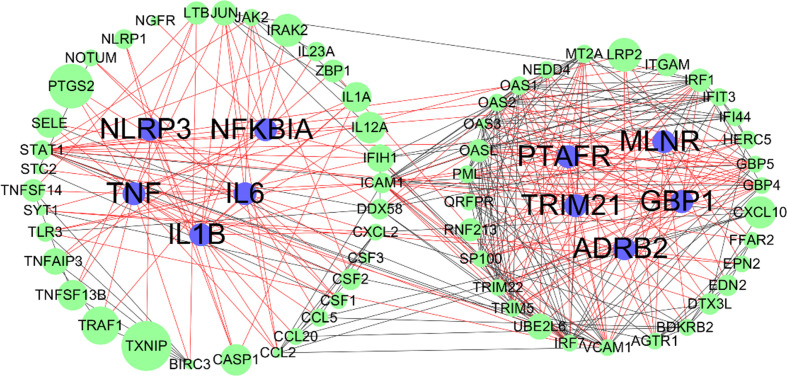
Protein-protein interaction (PPI) networks of SARS-CoV-2 differentially expressed genes (DEGs); blue nodes represent overlapping targets of Astragaloside IV and SARS-CoV-2; green nodes represent SARS-CoV-2 DEGs; edges represent correlations between targets, red edges represent overlapping targets related correlations, black edges represent SARS-CoV-2 DEGs related correlations; the size of the nodes indicates the value of combined score in STRING.

**Figure 7:**
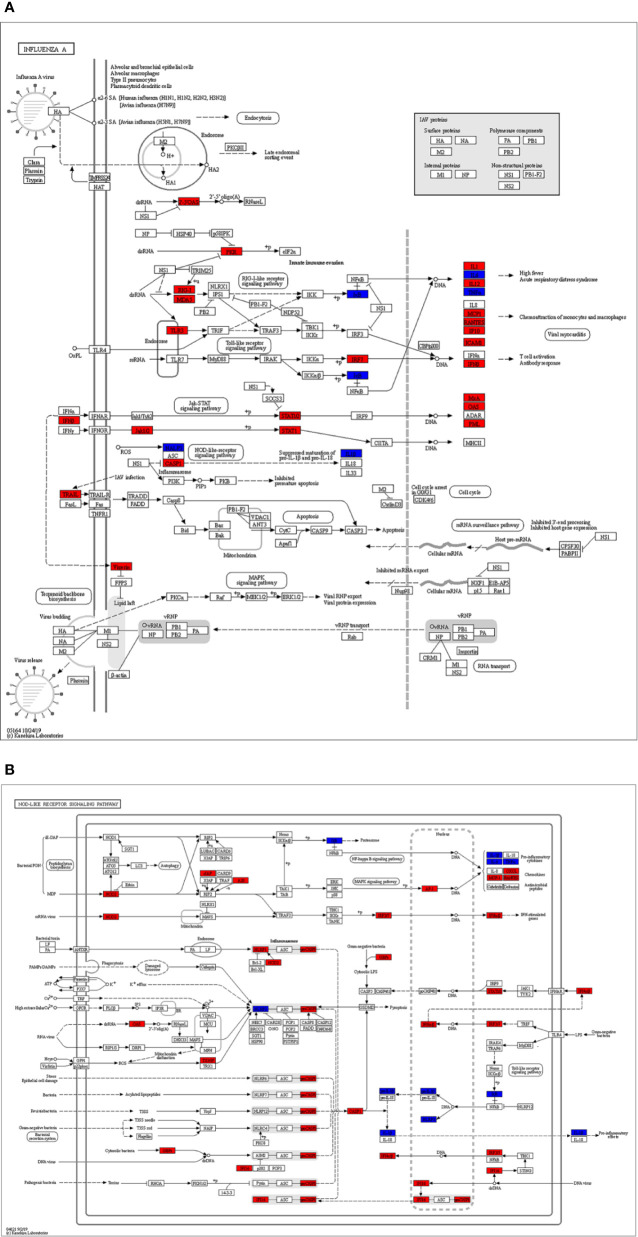
The identified key pathways. **(A)** Influenza A pathway. **(B)** NOD-like receptor signaling pathway. The red marked nodes represent up-regulated SARS-CoV-2 differentially expressed genes (DEGs), while the blue marked nodes represent overlapping targets of Astragaloside IV and SARS-CoV-2.

Overlapping targets of Astragaloside IV and SARS-CoV-2 DEGs included MMP13, NLRP3, TRIM21, GBP1, ADORA2A, PTAFR, TNF, MLNR, IL1B, NFKBIA, ADRB, and IL6. PPI network can visualize and quantify the function of specific proteins in cells at the systematic level (Jordan et al., 2012). Therefore, a PPI network of overlapping targets and SARS-CoV-2 DEGs was constructed and the common targets marked (**Figure 6**). The location of overlapping genes and SARS-CoV-2 DEGs in the key pathways are listed in **Figure 7**.

### Molecular Docking Analysis

**Figure 8:**
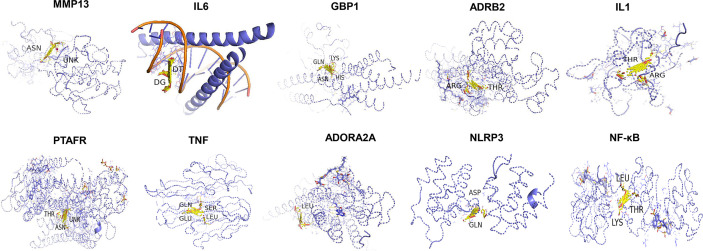
Structural interactions of Astragaloside IV and key target receptors.

Molecular docking analysis was conducted to evaluate the binding affinity of Astragaloside IV with key target receptors. The results showed that docking scores of Astragaloside IV with MMP13, NLRP3, GBP1, ADORA2A, PTAFR, TNF, MLNR, IL1B, NFKBIA, ADRB2, and IL6 ranged from −6.72 to −9.05. Particularly, Astragaloside IV presented the highest docking score with MMP13 and IL6 (docking score: −9.05, −9.04), demonstrating that Astragaloside IV is perfectly located inside the binding site together with MMP13 and IL6. Other key targets also showed an affinity with Astragaloside IV. Compared to Rapamycin, Astragaloside IV showed weaker binding affinities to these targets (**Figure 8**; **Table 2**).

**Table 2:** The docking scores of Astragaloside IV and Rapamycin with key proteins.

Target	PDB ID	Binding energy (kcal/mol)	
		Astragaloside IV	Rapamycin
MMP13	3I7I	−9.05	−16.27
IL6	4CNI	−9.04	−20.19
GBP1	6K1Z	−8.91	−13.8
ADRB2	3D4S	−8.18	−14.08
IL1	5BOW	−8.16	−17.84
PTAFR	5ZKQ	−7.84	−16.66
TNF	2ZJC	−7.78	−14.24
ADORA2A	2YDV	−7.13	−12.99
NLRP3	3QF2	−6.97	−12.11
NF-κB	6GJW	−6.72	−12.63

## Discussion

Astragaloside IV has been widely used in clinical practice (Qi et al., 2006; Xie et al., 2012; Yang et al., 2012). Pharmacokinetic properties of Astragaloside IV have been based on linear pharmacokinetics on doses ranging from 0.3mg/kg to 0.75mg/kg of Astragaloside injection. Astragaloside IV is safe and well-tolerated after intravenous infusion in clinical practice (Xu et al., 2013).

Studies have demonstrated that Astragaloside IV exerts anti-inflammatory effects *via* regulation of the NF-κB and JNK signaling pathway and inhibiting the release of inflammatory cytokines. Astragaloside IV inhibits the activation of NF-κB, by decreasing the phosphorylation of IκB and the nuclear translocation of NF-κB, thus downregulating the expression of TNF-α and IL-1β (Leng et al., 2018; Song et al., 2018; Wang et al., 2018; Leng et al., 2019). Astragaloside IV also inhibits adhesion-related molecules which are important in exerting protective anti-inflammatory effects. CD11b/CD18 is the key integrin on the surface of neutrophils. Astragaloside IV decreases the proportion of CD11b/CD18-positive neutrophils and reduces the expression of intercellular adhesion molecule-1 (ICAM-1), which is achieved by inhibiting the level of NF-κB and attenuating the expression of TNF-α and IL-1β (Li et al., 2012; Li et al., 2013).

Astragaloside IV has been widely in treating various inflammatory diseases caused by viruses. Astragaloside IV inhibits the replication of human adenovirus type 3 and apoptosis of A549 cells *in vitro*. The anti-virus properties are correlated with the concentration of astragaloside IV (Shang et al., 2011). Astragaloside IV exerts potential cardioprotective properties in viral myocarditis (Chen et al., 2011; Liu et al., 2019; Zhuang et al., 2019). Coxsackievirus B3 (CVB3) is an important pathogen for viral myocarditis. Astragaloside IV inhibits the proliferation of CVB3 by enhancing the expression of IFN-gamma (Zhang et al., 2006). In H1N1 Infection, Astragaloside IV inhibits IL-1β secretion by up-regulating Autophagy (Zhang J. et al., 2020).

Significantly up-regulated levels of cytokines and chemokines including IL1-β, IL1RA, IL10, IL9, IL8, IL7, basic FGF2, GCSF, GMCSF, IFNγ, IP10, MCP1, MIP1α, MIP1β, PDGFB, TNFα, and VEGFA in blood, have been confirmed in COVID-19 patients. The hyper-inflammation triggers the immune system to attack the body, and cause ARDS and multiple organ failure, and finally lead to death in severe cases of COVID-19 (Huang et al., 2020; Li et al., 2020; Rothan and Byrareddy, 2020). Therefore, there is an urgent need to find specific drugs or therapy for treating hyperinflammation in COVID-19.

In this study, a total of 425 potential Astragaloside IV related targets were screened from an online database. GO terms and KEGG pathways in which these targets enrich are consistent with the current understanding of Astragaloside IV(Ren et al., 2013; Li et al., 2017). Some of the upregulated SARS-CoV-2 DEGs included TNF, IL6, IL1A, IL1B, NLRP3, IL17C, IL12A. These results are consistent with current COVID-19 research, that there are elevated levels of cytokines in plasma of patients with COVID-19 (Huang et al., 2020).

The GO analysis results of SARS-CoV-2 DEGs revealed that type I interferon signaling pathway, cellular response to type I interferon, response to type I interferon, defense response to the virus, mRNA binding involved in posttranscriptional gene silencing were the most significant terms. Influenza A, Measles, NOD-like receptor signaling pathway, Hepatitis C, Herpes simplex infection, Cytokine-cytokine receptor interaction were the most enriched pathways in KEGG enrichment analysis. Hyper-activation of these pathways is involved in the SARS-CoV-2 infection, leading to the induction of a variety of pro-inflammatory cytokines, including IL-6, TNFα, and chemokines (Miossec and Kolls, 2012; Murakami et al., 2019; Xu et al., 2020). Inhibiting these signaling pathways to reduce the release of inflammatory factors may be key in the treatment of hyperinflammation in COVID-19. *NF-κB, IL-6, TNF* are considered to be therapeutic targets for COVID-19 (Mehta et al., 2020; Wang L. et al., 2020).

The overlapping targets of Astragaloside IV-related targets and SARS-CoV-2 DEGs can be considered as potential drug targets in the treatment of COVID-19. Astragaloside IV attenuates upstream nuclear translocation and phosphorylation of NF-κB-p65, which is a key component of the NF-κB pathway, and the activation of NF-κB leads to up-regulated expression of TNF-α and IL-1β (Liu et al., 2014).

Immunosuppressive drugs are likely to be beneficial in the treatment of COVID-19 infections. A study on IL-1 blockade (anakinra) in sepsis, showed a significant survival benefit in patients with hyperinflammation, without any apparent increased adverse events (Shakoory et al., 2016). Administration of IL-6 receptor blockade, licensed for cytokine release syndrome, has been approved in patients with COVID-19 pneumonia and elevated IL-6 in China (Liu et al., 2020; Zhang C. et al., 2020). Therefore, this study shows that the anti-inflammatory effects of astragaloside IV are sufficient to ameliorate a cytokine storm in the lungs, caused by COVID-19. However, these results need further experimental validation.

According to the molecular docking analysis results in our study, Astragaloside IV showed milder anti-inflammatory effects compared to standard pharmacological immunosuppressive drugs such as Rapamycin. Probably, it is less likely to affect the immune function and reduce the ability of the host to eliminate virus or bacteria. A large number of studies have confirmed that the pharmacological effects of Astragaloside IV are multi-level and multi-target (Ren et al., 2013; Li et al., 2017; Liu et al., 2019), which is beneficial to be a potential clinical therapeutic drug for COVID-19 considering the complex pathogenesis and complications.

In this study, the pharmacological mechanism of Astragaloside IV on COVID-19 is investigated using network pharmacology and bioinformatics analysis. Astragaloside IV is shown to be a potential drug for alleviating hyperinflammation in COVID-19 by inhibiting the Influenza A pathway, NOD-like receptor signaling pathway. Although direct evidence of Astragaloside IV application in COVID-19 is unclear, the anti-inflammatory effects of Astragaloside IV have been proven in numerous studies. Therefore, we believe that Astragaloside IV is likely to be beneficial in treating severe COVID-19 patients.

## Data Availability Statement

All datasets presented in this study are included in the article/**Supplementary Material**.

## Author Contributions

CG and YH contributed equally to this study. CG and YH participated in the design of this study and performed the statistical analysis. CG drafted the manuscript. All authors contributed to the article and approved the submitted version.

## Conflict of Interest

The authors declare that the research was conducted in the absence of any commercial or financial relationships that could be construed as a potential conflict of interest.
