# Supplementary material for: In Silico Prediction of Molecular Targets of Astragaloside IV for Alleviation of COVID-19 Hyperinflammation by Systems Network Pharmacology and Bioinformatic Gene Expression Analysis
Source: Front Pharmacol. 2020 Sep 16;11:556984. doi: 10.3389/fphar.2020.556984 (PMC7525161; doi:10.3389/fphar.2020.556984)
Supplement: Supplementary file 1 [file Table_1.docx]

**Table S1: Potential targets of Astragaloside IV**

| **Target** | **Description** | **Database** |
| --- | --- | --- |
| **ATP2A2** | ATPase sarcoplasmic/endoplasmic reticulum Ca2+ transporting 2 | CTD |
| **BAX** | BCL2 associated X, apoptosis regulator | CTD |
| **BCS1L** | BCS1 homolog, ubiquinol-cytochrome c reductase complex chaperone | CTD |
| **CASP3** | caspase 3 | CTD |
| **GPT** | glutamic--pyruvic transaminase | CTD |
| **HMOX1** | heme oxygenase 1 | CTD |
| **IL1B** | interleukin 1 beta | CTD |
| **IL6** | interleukin 6 | CTD |
| **MPO** | myeloperoxidase | CTD |
| **NFE2L2** | nuclear factor, erythroid 2 like 2 | CTD |
| **NFKBIA** | NFKB inhibitor alpha | CTD |
| **NFKBIB** | NFKB inhibitor beta | CTD |
| **NLRP3** | NLR family pyrin domain containing 3 | CTD |
| **PLN** | phospholamban | CTD |
| **RELA** | RELA proto-oncogene, NF-kB subunit | CTD |
| **SOD1** | superoxide dismutase 1 | CTD |
| **SOD2** | superoxide dismutase 2 | CTD |
| **SQSTM1** | sequestosome 1 | CTD |
| **TNF** | tumor necrosis factor | CTD |
| **SLC5A2** | solute carrier family 5 member 2 | TargetNet |
| **SLC5A1** | solute carrier family 5 member 1 | TargetNet |
| **NR2F2** | nuclear receptor subfamily 2 group F member 2 | TargetNet |
| **GALR3** | galanin receptor 3 | TargetNet |
| **ache** | acetylcholinesterase (Cartwright blood group) | TargetNet |
| **MMP13** | matrix metallopeptidase 13 | PharmMapper |
| **NLRP3** | NLR family pyrin domain containing 3 | PharmMapper |
| **TRIM21** | tripartite motif containing 21 | PharmMapper |
| **GBP1** | guanylate binding protein 1 | PharmMapper |
| **ADORA2A** | adenosine A2a receptor | PharmMapper |
| **PTAFR** | platelet activating factor receptor | PharmMapper |
| **TNF** | tumor necrosis factor | PharmMapper |
| **MLNR** | motilin receptor | PharmMapper |
| **IL1B** | interleukin 1 beta | PharmMapper |
| **NFKBIA** | NFKB inhibitor alpha | PharmMapper |
| **ADRB2** | adrenoceptor beta 2 | PharmMapper |
| **IL6** | interleukin 6 | PharmMapper |
| **LAP3** | leucine aminopeptidase 3 | PharmMapper |
| **CAM** | calmodulin 1 | PharmMapper |
| **NR1D2** | nuclear receptor subfamily 1 group D member 2 | PharmMapper |
| **aldA** | aldolase, fructose-bisphosphate A | PharmMapper |
| **sdhA** | succinate dehydrogenase complex flavoprotein subunit A | PharmMapper |
| **ahcY** | adenosylhomocysteinase | PharmMapper |
| **SDHA** | succinate dehydrogenase complex flavoprotein subunit A | PharmMapper |
| **CITED2** | Cbp/p300 interacting transactivator with Glu/Asp rich carboxy-terminal domain 2 | PharmMapper |
| **frdA** | frataxin | PharmMapper |
| **RGS6** | regulator of G protein signaling 6 | PharmMapper |
| **Capn2** | calpain 2 | PharmMapper |
| **Edc3** | enhancer of mRNA decapping 3 | PharmMapper |
| **SYNCRIP** | synaptotagmin binding cytoplasmic RNA interacting protein | PharmMapper |
| **Flot2** | flotillin 2 | PharmMapper |
| **ARHGAP11A** | Rho GTPase activating protein 11A | PharmMapper |
| **USP14** | ubiquitin specific peptidase 14 | PharmMapper |
| **Ache** | acetylcholinesterase (Cartwright blood group) | PharmMapper |
| **TNNC2** | troponin C2, fast skeletal type | PharmMapper |
| **POT1** | protection of telomeres 1 | PharmMapper |
| **PDI1** | peptidyl arginine deiminase 1 | PharmMapper |
| **CSDE1** | cold shock domain containing E1 | PharmMapper |
| **caf1** | chromatin assembly factor 1 subunit A | PharmMapper |
| **Sds** | serine dehydratase | PharmMapper |
| **RNR2** | l-rRNA | PharmMapper |
| **ARHGEF12** | Rho guanine nucleotide exchange factor 12 | PharmMapper |
| **PLA2G2A** | phospholipase A2 group IIA | PharmMapper |
| **oprM** | opioid receptor mu 1 | PharmMapper |
| **FES** | FES proto-oncogene, tyrosine kinase | PharmMapper |
| **HSD17B4** | hydroxysteroid 17-beta dehydrogenase 4 | PharmMapper |
| **NPC2** | NPC intracellular cholesterol transporter 2 | PharmMapper |
| **RARB** | retinoic acid receptor beta | PharmMapper |
| **PMP2** | peripheral myelin protein 2 | PharmMapper |
| **UAP1** | UDP-N-acetylglucosamine pyrophosphorylase 1 | PharmMapper |
| **hemB** | coagulation factor IX | PharmMapper |
| **Kcnip3** | potassium voltage-gated channel interacting protein 3 | PharmMapper |
| **GBP1** | guanylate binding protein 1 | PharmMapper |
| **Crygs** | crystallin gamma S | PharmMapper |
| **RAB31** | RAB31, member RAS oncogene family | PharmMapper |
| **ACTA1** | actin alpha 1, skeletal muscle | PharmMapper |
| **CAMK4** | calcium/calmodulin dependent protein kinase IV | PharmMapper |
| **hbp** | stem-loop binding protein | PharmMapper |
| **MEF2A** | myocyte enhancer factor 2A | PharmMapper |
| **Trpv6** | transient receptor potential cation channel subfamily V member 6 | PharmMapper |
| **ppaC** | cellular communication network factor 6 | PharmMapper |
| **RAB7A** | RAB7A, member RAS oncogene family | PharmMapper |
| **pac** | proton activated chloride channel 1 | PharmMapper |
| **CDC4** | F-box and WD repeat domain containing 7 | PharmMapper |
| **trpA** | tryptase gamma 1 | PharmMapper |
| **USP19** | ubiquitin specific peptidase 19 | PharmMapper |
| **PIP4K2C** | phosphatidylinositol-5-phosphate 4-kinase type 2 gamma | PharmMapper |
| **icd** | N-acetylglucosamine-1-phosphate transferase subunits alpha and beta | PharmMapper |
| **TAF13** | TATA-box binding protein associated factor 13 | PharmMapper |
| **HSD17B1** | hydroxysteroid 17-beta dehydrogenase 1 | PharmMapper |
| **clpX** | caseinolytic mitochondrial matrix peptidase chaperone subunit X | PharmMapper |
| **Onecut1** | one cut homeobox 1 | PharmMapper |
| **Anxa5** | annexin A5 | PharmMapper |
| **ssb** | small RNA binding exonuclease protection factor La | PharmMapper |
| **CCNE1** | cyclin E1 | PharmMapper |
| **MAPK9** | mitogen-activated protein kinase 9 | PharmMapper |
| **HBA** | sodium voltage-gated channel alpha subunit 2 | PharmMapper |
| **srb11** | cyclin C | PharmMapper |
| **GAD1** | glutamate decarboxylase 1 | PharmMapper |
| **AZGP1** | alpha-2-glycoprotein 1, zinc-binding | PharmMapper |
| **Gpi** | glucose-6-phosphate isomerase | PharmMapper |
| **ndkA** | NME/NM23 nucleoside diphosphate kinase 1 | PharmMapper |
| **USH1C** | USH1 protein network component harmonin | PharmMapper |
| **Sin3b** | SIN3 transcription regulator family member B | PharmMapper |
| **for** | WW domain containing oxidoreductase | PharmMapper |
| **ARHGAP5** | Rho GTPase activating protein 5 | PharmMapper |
| **S100B** | S100 calcium binding protein B | PharmMapper |
| **S100A6** | S100 calcium binding protein A6 | PharmMapper |
| **ACR** | acrosin | PharmMapper |
| **fabD** | malonyl-CoA-acyl carrier protein transacylase | PharmMapper |
| **DGCR8** | DGCR8 microprocessor complex subunit | PharmMapper |
| **ACBD7** | acyl-CoA binding domain containing 7 | PharmMapper |
| **MS4A1** | membrane spanning 4-domains A1 | PharmMapper |
| **Tp53** | tumor protein p53 | PharmMapper |
| **CBL** | Cbl proto-oncogene | PharmMapper |
| **Ubl5** | ubiquitin like 5 | PharmMapper |
| **cgi121** | TP53RK binding protein | PharmMapper |
| **PRDX6** | peroxiredoxin 6 | PharmMapper |
| **MB** | myoglobin | PharmMapper |
| **S100a13** | S100 calcium binding protein A13 | PharmMapper |
| **LSS** | lanosterol synthase | PharmMapper |
| **alr** | lysine methyltransferase 2D | PharmMapper |
| **Sec15** | exocyst complex component 6 | PharmMapper |
| **HDAC7** | histone deacetylase 7 | PharmMapper |
| **Adh** | arginine vasopressin | PharmMapper |
| **TIMM9** | translocase of inner mitochondrial membrane 9 | PharmMapper |
| **HBB** | hemoglobin subunit beta | PharmMapper |
| **dld** | dihydrolipoamide dehydrogenase | PharmMapper |
| **Lyn** | LYN proto-oncogene, Src family tyrosine kinase | PharmMapper |
| **cat** | catalase | PharmMapper |
| **Gmnn** | geminin DNA replication inhibitor | PharmMapper |
| **trkA** | neurotrophic receptor tyrosine kinase 1 | PharmMapper |
| **Mog** | myelin oligodendrocyte glycoprotein | PharmMapper |
| **MMP2** | matrix metallopeptidase 2 | PharmMapper |
| **fpr** | formyl peptide receptor 1 | PharmMapper |
| **GYG1** | glycogenin 1 | PharmMapper |
| **COG2** | component of oligomeric golgi complex 2 | PharmMapper |
| **mgsA** | C-X-C motif chemokine ligand 1 | PharmMapper |
| **STARD5** | StAR related lipid transfer domain containing 5 | PharmMapper |
| **nadE** | brain expressed X-linked 3 | PharmMapper |
| **cat3** | solute carrier family 7 member 3 | PharmMapper |
| **spt16** | SPT16 homolog, facilitates chromatin remodeling subunit | PharmMapper |
| **Vdr** | vitamin D receptor | PharmMapper |
| **ZBTB21** | zinc finger and BTB domain containing 21 | PharmMapper |
| **ANXA1** | annexin A1 | PharmMapper |
| **ITPKC** | inositol-trisphosphate 3-kinase C | PharmMapper |
| **GALM** | galactose mutarotase | PharmMapper |
| **era** | estrogen receptor 1 | PharmMapper |
| **GDI1** | GDP dissociation inhibitor 1 | PharmMapper |
| **hba1** | hemoglobin subunit alpha 1 | PharmMapper |
| **NMRK1** | nicotinamide riboside kinase 1 | PharmMapper |
| **NR3C2** | nuclear receptor subfamily 3 group C member 2 | PharmMapper |
| **MUC1** | mucin 1, cell surface associated | PharmMapper |
| **RDM1** | RAD52 motif containing 1 | PharmMapper |
| **PAP2** | MYB binding protein 1a | PharmMapper |
| **PDK3** | pyruvate dehydrogenase kinase 3 | PharmMapper |
| **AR** | androgen receptor | PharmMapper |
| **SUB1** | SUB1 regulator of transcription | PharmMapper |
| **DTYMK** | deoxythymidylate kinase | PharmMapper |
| **Cd2** | CD2 molecule | PharmMapper |
| **WARS1** | tryptophanyl-tRNA synthetase 1 | PharmMapper |
| **Fabp2** | fatty acid binding protein 2 | PharmMapper |
| **KYNU** | kynureninase | PharmMapper |
| **VAV3** | vav guanine nucleotide exchange factor 3 | PharmMapper |
| **AKR1C4** | aldo-keto reductase family 1 member C4 | PharmMapper |
| **HDAC6** | histone deacetylase 6 | PharmMapper |
| **NR1I3** | nuclear receptor subfamily 1 group I member 3 | PharmMapper |
| **CRS2** | msh homeobox 2 | PharmMapper |
| **rho** | rhodopsin | PharmMapper |
| **def** | UTP25 small subunit processor component | PharmMapper |
| **GAL10** | Charcot-Leyden crystal galectin | PharmMapper |
| **shp** | nuclear receptor subfamily 0 group B member 2 | PharmMapper |
| **CAM1** | calmodulin 2 | PharmMapper |
| **MAGEA4** | MAGE family member A4 | PharmMapper |
| **IRF4** | interferon regulatory factor 4 | PharmMapper |
| **MSN** | moesin | PharmMapper |
| **TRIM21** | tripartite motif containing 21 | PharmMapper |
| **MYO6** | myosin VI | PharmMapper |
| **SRP54** | signal recognition particle 54 | PharmMapper |
| **HSP90AA1** | heat shock protein 90 alpha family class A member 1 | SwissTargetPrediction |
| **VEGFA** | vascular endothelial growth factor A | SwissTargetPrediction |
| **FGF1** | fibroblast growth factor 1 | SwissTargetPrediction |
| **FGF2** | fibroblast growth factor 2 | SwissTargetPrediction |
| **HPSE** | heparanase | SwissTargetPrediction |
| **CDK1** | cyclin dependent kinase 1 | SwissTargetPrediction |
| **LGALS4** | galectin 4 | SwissTargetPrediction |
| **LGALS3** | galectin 3 | SwissTargetPrediction |
| **LGALS8** | galectin 8 | SwissTargetPrediction |
| **HTR2B** | 5-hydroxytryptamine receptor 2B | SwissTargetPrediction |
| **ADRA2A** | adrenoceptor alpha 2A | SwissTargetPrediction |
| **ADRA2C** | adrenoceptor alpha 2C | SwissTargetPrediction |
| **ADRA2B** | adrenoceptor alpha 2B | SwissTargetPrediction |
| **DRD1** | dopamine receptor D1 | SwissTargetPrediction |
| **ADRA1D** | adrenoceptor alpha 1D | SwissTargetPrediction |
| **HTR2C** | 5-hydroxytryptamine receptor 2C | SwissTargetPrediction |
| **CYP2D6** | cytochrome P450 family 2 subfamily D member 6 | SwissTargetPrediction |
| **HTR6** | 5-hydroxytryptamine receptor 6 | SwissTargetPrediction |
| **ADRA1A** | adrenoceptor alpha 1A | SwissTargetPrediction |
| **HTR1B** | 5-hydroxytryptamine receptor 1B | SwissTargetPrediction |
| **STAT3** | signal transducer and activator of transcription 3 | SwissTargetPrediction |
| **AKT2** | AKT serine/threonine kinase 2 | SwissTargetPrediction |
| **RPS6KA1** | ribosomal protein S6 kinase A1 | SwissTargetPrediction |
| **ROCK1** | Rho associated coiled-coil containing protein kinase 1 | SwissTargetPrediction |
| **AKT1** | AKT serine/threonine kinase 1 | SwissTargetPrediction |
| **TRPV1** | transient receptor potential cation channel subfamily V member 1 | SwissTargetPrediction |
| **CNR1** | cannabinoid receptor 1 | SwissTargetPrediction |
| **CNR2** | cannabinoid receptor 2 | SwissTargetPrediction |
| **PTAFR** | platelet activating factor receptor | SwissTargetPrediction |
| **TYMS** | thymidylate synthetase | SwissTargetPrediction |
| **ADORA1** | adenosine A1 receptor | SwissTargetPrediction |
| **ADORA2A** | adenosine A2a receptor | SwissTargetPrediction |
| **OPRK1** | opioid receptor kappa 1 | SwissTargetPrediction |
| **FAAH** | fatty acid amide hydrolase | SwissTargetPrediction |
| **SLC6A2** | solute carrier family 6 member 2 | SwissTargetPrediction |
| **ADRB2** | adrenoceptor beta 2 | SwissTargetPrediction |
| **BCL2L1** | BCL2 like 1 | SwissTargetPrediction |
| **GLRA1** | glycine receptor alpha 1 | SwissTargetPrediction |
| **GLRA2** | glycine receptor alpha 2 | SwissTargetPrediction |
| **MTOR** | mechanistic target of rapamycin kinase | SwissTargetPrediction |
| **PIK3CG** | phosphatidylinositol-4,5-bisphosphate 3-kinase catalytic subunit gamma | SwissTargetPrediction |
| **PIK3CA** | phosphatidylinositol-4,5-bisphosphate 3-kinase catalytic subunit alpha | SwissTargetPrediction |
| **IL2** | interleukin 2 | SwissTargetPrediction |
| **RORC** | RAR related orphan receptor C | SwissTargetPrediction |
| **MDM2** | MDM2 proto-oncogene | SwissTargetPrediction |
| **SF3B3** | splicing factor 3b subunit 3 | SwissTargetPrediction |
| **S1PR1** | sphingosine-1-phosphate receptor 1 | SwissTargetPrediction |
| **ADRB1** | adrenoceptor beta 1 | SwissTargetPrediction |
| **F10** | coagulation factor X | SwissTargetPrediction |
| **SSTR5** | somatostatin receptor 5 | SwissTargetPrediction |
| **SSTR2** | somatostatin receptor 2 | SwissTargetPrediction |
| **SSTR4** | somatostatin receptor 4 | SwissTargetPrediction |
| **SSTR1** | somatostatin receptor 1 | SwissTargetPrediction |
| **SSTR3** | somatostatin receptor 3 | SwissTargetPrediction |
| **SRC** | SRC proto-oncogene, non-receptor tyrosine kinase | SwissTargetPrediction |
| **PPM1A** | protein phosphatase, Mg2+/Mn2+ dependent 1A | SwissTargetPrediction |
| **IGF2R** | insulin like growth factor 2 receptor | SwissTargetPrediction |
| **OPRD1** | opioid receptor delta 1 | SwissTargetPrediction |
| **DRD2** | dopamine receptor D2 | SwissTargetPrediction |
| **HTR2A** | 5-hydroxytryptamine receptor 2A | SwissTargetPrediction |
| **DRD3** | dopamine receptor D3 | SwissTargetPrediction |
| **F11** | coagulation factor XI | SwissTargetPrediction |
| **F7** | coagulation factor VII | SwissTargetPrediction |
| **LNPEP** | leucyl and cystinyl aminopeptidase | SwissTargetPrediction |
| **ERAP2** | endoplasmic reticulum aminopeptidase 2 | SwissTargetPrediction |
| **ERAP1** | endoplasmic reticulum aminopeptidase 1 | SwissTargetPrediction |
| **VDR** | vitamin D receptor | SwissTargetPrediction |
| **PIK3CD** | phosphatidylinositol-4,5-bisphosphate 3-kinase catalytic subunit delta | SwissTargetPrediction |
| **PTPN2** | protein tyrosine phosphatase non-receptor type 2 | SwissTargetPrediction |
| **PTPRA** | protein tyrosine phosphatase receptor type A | SwissTargetPrediction |
| **TACR2** | tachykinin receptor 2 | SwissTargetPrediction |
| **CXCR3** | C-X-C motif chemokine receptor 3 | SwissTargetPrediction |
| **EGFR** | epidermal growth factor receptor | SwissTargetPrediction |
| **ATP1A1** | ATPase Na+/K+ transporting subunit alpha 1 | SwissTargetPrediction |
| **MGAM** | maltase-glucoamylase | SwissTargetPrediction |
| **SI** | sucrase-isomaltase | SwissTargetPrediction |
| **SLC10A2** | solute carrier family 10 member 2 | SwissTargetPrediction |
| **PTPN11** | protein tyrosine phosphatase non-receptor type 11 | SwissTargetPrediction |
| **MLNR** | motilin receptor | SwissTargetPrediction |
| **MMP13** | matrix metallopeptidase 13 | SwissTargetPrediction |
| **MMP1** | matrix metallopeptidase 1 | SwissTargetPrediction |
| **ADAM17** | ADAM metallopeptidase domain 17 | SwissTargetPrediction |
| **HTR1A** | 5-hydroxytryptamine receptor 1A | SwissTargetPrediction |
| **SLC6A4** | solute carrier family 6 member 4 | SwissTargetPrediction |
| **SELL** | selectin L | SwissTargetPrediction |
| **SELP** | selectin P | SwissTargetPrediction |
| **GBA** | glucosylceramidase beta | SwissTargetPrediction |
| **FPGS** | folylpolyglutamate synthase | SwissTargetPrediction |
| **ABCB1** | ATP binding cassette subfamily B member 1 | SwissTargetPrediction |
| **EGLN2** | egl-9 family hypoxia inducible factor 2 | SwissTargetPrediction |
| **EGLN1** | egl-9 family hypoxia inducible factor 1 | SwissTargetPrediction |
| **EGLN3** | egl-9 family hypoxia inducible factor 3 | SwissTargetPrediction |
| **TYR** | tyrosinase | SwissTargetPrediction |
| **CA12** | carbonic anhydrase 12 | SwissTargetPrediction |
| **CA9** | carbonic anhydrase 9 | SwissTargetPrediction |
| **FDFT1** | farnesyl-diphosphate farnesyltransferase 1 | SwissTargetPrediction |
| **PSEN2** | presenilin 2 | SwissTargetPrediction |
| **PSENEN** | presenilin enhancer, gamma-secretase subunit | SwissTargetPrediction |
| **NCSTN** | nicastrin | SwissTargetPrediction |
| **APH1A** | aph-1 homolog A, gamma-secretase subunit | SwissTargetPrediction |
| **PSEN1** | presenilin 1 | SwissTargetPrediction |
| **APH1B** | aph-1 homolog B, gamma-secretase subunit | SwissTargetPrediction |
| **ITGB3** | integrin subunit beta 3 | SwissTargetPrediction |
| **PIK3R1** | phosphoinositide-3-kinase regulatory subunit 1 | SwissTargetPrediction |
