# Supplementary material for: In Silico Prediction of Molecular Targets of Astragaloside IV for Alleviation of COVID-19 Hyperinflammation by Systems Network Pharmacology and Bioinformatic Gene Expression Analysis
Source: Front Pharmacol. 2020 Sep 16;11:556984. doi: 10.3389/fphar.2020.556984 (PMC7525161; doi:10.3389/fphar.2020.556984)
Supplement: Supplementary file 2 [file Table_2.docx]

**Table S2: Differentially** **expressed** **genes** **(DEGs) of SARS-CoV-2**

| **Gene Symbol** | **Description** | **logFC** | **adj.P.Val** |
| --- | --- | --- | --- |
| **ISG15** | ISG15 ubiquitin like modifier | 3.770162 | 0.001132 |
| **IFI6** | interferon alpha inducible protein 6 | 4.284894 | 0.001368 |
| **MX1** | MX dynamin like GTPase 1 | 5.004951 | 0.001368 |
| **IFIT1** | interferon induced protein with tetratricopeptide repeats 1 | 4.305935 | 0.001368 |
| **IRF9** | interferon regulatory factor 9 | 2.117787 | 0.001888 |
| **PARP9** | poly(ADP-ribose) polymerase family member 9 | 2.038141 | 0.0024 |
| **OAS1** | 2'-5'-oligoadenylate synthetase 1 | 1.527643 | 0.00362 |
| **IRF7** | interferon regulatory factor 7 | 3.194253 | 0.003769 |
| **OAS2** | 2'-5'-oligoadenylate synthetase 2 | 4.292692 | 0.003782 |
| **IFIT3** | interferon induced protein with tetratricopeptide repeats 3 | 1.955261 | 0.003782 |
| **DDX60** | DExD/H-box helicase 60 | 2.274155 | 0.004099 |
| **OAS3** | 2'-5'-oligoadenylate synthetase 3 | 1.365455 | 0.00972 |
| **DTX3L** | deltex E3 ubiquitin ligase 3L | 1.350932 | 0.013657 |
| **SP110** | SP110 nuclear body protein | 1.24423 | 0.013993 |
| **IFITM1** | interferon induced transmembrane protein 1 | 3.965357 | 0.003769 |
| **HELZ2** | helicase with zinc finger 2 | 1.469536 | 0.019567 |
| **HERC6** | HECT and RLD domain containing E3 ubiquitin protein ligase family member 6 | 1.446734 | 0.019924 |
| **STAT1** | signal transducer and activator of transcription 1 | 1.288918 | 0.019924 |
| **IFIH1** | interferon induced with helicase C domain 1 | 1.827547 | 0.022323 |
| **PARP10** | poly(ADP-ribose) polymerase family member 10 | 1.878133 | 0.030676 |
| **ZAR1** | zygote arrest 1 | -1.49279 | 0.019567 |
| **DDX58** | DExD/H-box helicase 58 | 1.739129 | 0.045009 |
| **IFI35** | interferon induced protein 35 | 1.111803 | 0.04774 |
| **CMPK2** | cytidine/uridine monophosphate kinase 2 | 2.227557 | 0.04774 |
| **PLSCR1** | phospholipid scramblase 1 | 1.059262 | 0.04774 |
| **TRIM14** | tripartite motif containing 14 | 1.111004 | 0.04774 |
| **SNORD26** | small nucleolar RNA, C/D box 26 | 2.271148 | 0.04774 |
| **LOC158435** | uncharacterized LOC158435 | -2.60608 | 0.048121 |
| **MYO7B** | myosin VIIB | -1.51083 | 0.0498 |
| **IFI27** | interferon alpha inducible protein 27 | 3.524604 | 0.061682 |
| **REC8** | REC8 meiotic recombination protein | 1.393842 | 0.062144 |
| **SERPINB7** | serpin family B member 7 | 1.155493 | 0.066459 |
| **PRR18** | proline rich 18 | -1.46135 | 0.070164 |
| **EIF2AK2** | eukaryotic translation initiation factor 2 alpha kinase 2 | 1.203579 | 0.076031 |
| **HSD11B1** | hydroxysteroid 11-beta dehydrogenase 1 | 1.549947 | 0.09013 |
| **THEMIS2** | thymocyte selection associated family member 2 | 1.690859 | 0.09013 |
| **TICAM2** | toll like receptor adaptor molecule 2 | -1.12359 | 0.09013 |
| **OASL** | 2'-5'-oligoadenylate synthetase like | 2.636842 | 0.090133 |
| **CFB** | complement factor B | 1.074856 | 0.099946 |
| **ST7-AS1** | ST7 antisense RNA 1 | 1.529803 | 0.10426 |
| **OTUD7A** | OTU deubiquitinase 7A | -1.04267 | 0.105622 |
| **EPSTI1** | epithelial stromal interaction 1 | 3.028684 | 0.040998 |
| **BATF2** | basic leucine zipper ATF-like transcription factor 2 | 1.346777 | 0.107611 |
| **MIR4444-1** | microRNA 4444-1 | 1.502223 | 0.084558 |
| **C2CD4C** | C2 calcium dependent domain containing 4C | 1.050083 | 0.111824 |
| **SERPINB3** | serpin family B member 3 | 2.311477 | 0.123172 |
| **MMP10** | matrix metallopeptidase 10 | 1.235622 | 0.123172 |
| **FCER1G** | Fc fragment of IgE receptor Ig | 1.873712 | 0.084558 |
| **CCL20** | C-C motif chemokine ligand 20 | 1.004091 | 0.128628 |
| **TRABD2B** | TraB domain containing 2B | -1.61227 | 0.128628 |
| **C22orf24** | chromosome 22 open reading frame 24 | 1.278174 | 0.134231 |
| **CSF3** | colony stimulating factor 3 | 1.409321 | 0.129622 |
| **GBP1** | guanylate binding protein 1 | 1.059237 | 0.131141 |
| **KRT4** | keratin 4 | -1.15915 | 0.134231 |
| **PSG9** | pregnancy specific beta-1-glycoprotein 9 | -2.7187 | 0.134231 |
| **SAMD9L** | sterile alpha motif domain containing 9 like | 1.971183 | 0.134231 |
| **IL1B** | interleukin 1 beta | 1.026088 | 0.142782 |
| **RPS15AP10** | ribosomal protein S15a pseudogene 10 | -2.36848 | 0.142993 |
| **MIR3176** | microRNA 3176 | -1.53097 | 0.142993 |
| **RSAD2** | radical S-adenosyl methionine domain containing 2 | 1.828237 | 0.142993 |
| **DISC1** | DISC1 scaffold protein | -1.51579 | 0.17505 |
| **FLJ26850** | None | -1.51579 | 0.17505 |
| **ACPP** | acid phosphatase 3 | -1.51579 | 0.17505 |
| **RAI2** | retinoic acid induced 2 | -1.51579 | 0.17505 |
| **EIF1B-AS1** | EIF1B antisense RNA 1 | -1.76891 | 0.178095 |
| **IFI44** | interferon induced protein 44 | 4.543779 | 0.178095 |
| **EGR1** | early growth response 1 | 1.022469 | 0.17505 |
| **MAGOH2** | mago homolog 2, pseudogene | -1.70593 | 0.183768 |
| **LOC100289495** | uncharacterized LOC100289495 | 1.050198 | 0.198195 |
| **IFIT2** | interferon induced protein with tetratricopeptide repeats 2 | 1.220931 | 0.189113 |
| **SNORD19** | small nucleolar RNA, C/D box 19 | 2.639994 | 0.198195 |
| **PDE1A** | phosphodiesterase 1A | 1.088238 | 0.197387 |
| **HSP90B2P** | heat shock protein 90 beta family member 2, pseudogene | 1.021085 | 0.198195 |
| **TDRD5** | tudor domain containing 5 | 1.040634 | 0.207689 |
| **CGB2** | chorionic gonadotropin subunit beta 2 | -1.79248 | 0.235496 |
| **CNTN2** | contactin 2 | -1.37744 | 0.237615 |
| **CMAHP** | cytidine monophospho-N-acetylneuraminic acid hydroxylase, pseudogene | -1.37744 | 0.237615 |
| **PURG** | purine rich element binding protein G | -1.37744 | 0.237615 |
| **CXCL10** | C-X-C motif chemokine ligand 10 | 2.721426 | 0.237615 |
| **FLJ37201** | tigger transposable element derived 2 pseudogene | 1.337963 | 0.228417 |
| **CCDC17** | coiled-coil domain containing 17 | -1.06545 | 0.228417 |
| **SAMD9** | sterile alpha motif domain containing 9 | 1.706923 | 0.228649 |
| **ROBO4** | roundabout guidance receptor 4 | -1.72331 | 0.239479 |
| **PMCH** | pro-melanin concentrating hormone | -1.72331 | 0.239479 |
| **GNRHR** | gonadotropin releasing hormone receptor | -1.72331 | 0.239479 |
| **PCDHA10** | protocadherin alpha 10 | -1.72331 | 0.239479 |
| **UBXN10** | UBX domain protein 10 | 1.276574 | 0.235496 |
| **KCNJ2** | potassium inwardly rectifying channel subfamily J member 2 | 1.375603 | 0.237615 |
| **CLEC18C** | C-type lectin domain family 18 member C | -2.77537 | 0.259409 |
| **NTSR1** | neurotensin receptor 1 | -2.31871 | 0.228417 |
| **LRRTM2** | leucine rich repeat transmembrane neuronal 2 | -2.31871 | 0.228417 |
| **C9orf139** | chromosome 9 open reading frame 139 | -2.31871 | 0.228417 |
| **C1orf229** | chromosome 1 open reading frame 229 | -2.89793 | 0.235496 |
| **MYO1H** | myosin IH | -1.23211 | 0.237615 |
| **EPHA7** | EPH receptor A7 | 1.857123 | 0.237615 |
| **TCAM1P** | testicular cell adhesion molecule 1, pseudogene | -3.3368 | 0.237615 |
| **MIR130B** | microRNA 130b | -1.72784 | 0.239479 |
| **MMEL1** | membrane metalloendopeptidase like 1 | -1.58496 | 0.239479 |
| **LINC00622** | long intergenic non-protein coding RNA 622 | -1.58496 | 0.239479 |
| **ADRA2A** | adrenoceptor alpha 2A | -1.58496 | 0.239479 |
| **MKRN9P** | makorin ring finger protein 9, pseudogene | -1.58496 | 0.239479 |
| **CCDC63** | coiled-coil domain containing 63 | -1.58496 | 0.239479 |
| **RRN3P2** | RRN3 pseudogene 2 | -1.58496 | 0.239479 |
| **SLFN12** | schlafen family member 12 | -1.58496 | 0.239479 |
| **SNORD1B** | small nucleolar RNA, C/D box 1B | -1.58496 | 0.239479 |
| **C9orf92** | chromosome 9 open reading frame 92 | -1.58496 | 0.239479 |
| **APOA1** | apolipoprotein A1 | -1.58496 | 0.273824 |
| **GPR111** | adhesion G protein-coupled receptor F2 | -1.58496 | 0.273824 |
| **CCDC158** | coiled-coil domain containing 158 | -2.1112 | 0.2405 |
| **DMD** | dystrophin | -2.1112 | 0.2405 |
| **BPIFB1** | BPI fold containing family B member 1 | -1.62886 | 0.259409 |
| **CLDN14** | claudin 14 | -1.00051 | 0.239479 |
| **C1orf189** | chromosome 1 open reading frame 189 | -1.90368 | 0.257053 |
| **SMTNL1** | smoothelin like 1 | 1.557475 | 0.239479 |
| **NDNF** | neuron derived neurotrophic factor | -1.47393 | 0.259409 |
| **RANBP3L** | RAN binding protein 3 like | -2.85137 | 0.262767 |
| **SERPINB4** | serpin family B member 4 | 2.150404 | 0.240347 |
| **NHLRC4** | NHL repeat containing 4 | -1.93578 | 0.284042 |
| **COL18A1-AS2** | COL18A1 antisense RNA 2 | -1.93578 | 0.284042 |
| **MIR4746** | microRNA 4746 | -2.16096 | 0.287803 |
| **PLEKHA4** | pleckstrin homology domain containing A4 | 1.08671 | 0.257053 |
| **SAA1** | serum amyloid A1 | 1.107841 | 0.258099 |
| **CLVS1** | clavesin 1 | 1.570033 | 0.259409 |
| **CCDC8** | coiled-coil domain containing 8 | -1.69616 | 0.300183 |
| **AQP4** | aquaporin 4 | -1.63952 | 0.289238 |
| **RCAN3AS** | RCAN3 antisense RNA | -2.6438 | 0.302744 |
| **CCR6** | C-C motif chemokine receptor 6 | -2.6438 | 0.302744 |
| **UOX** | urate oxidase (pseudogene) | -1.16993 | 0.302744 |
| **RTL1** | retrotransposon Gag like 1 | -1.16993 | 0.302744 |
| **SPNS3** | sphingolipid transporter 3 (putative) | 1.169925 | 0.302744 |
| **FUT1** | fucosyltransferase 1 (H blood group) | 1.169925 | 0.302744 |
| **STON1-GTF2A1L** | STON1-GTF2A1L readthrough | -1.16993 | 0.302744 |
| **SULT1C4** | sulfotransferase family 1C member 4 | 1.169925 | 0.302744 |
| **GALR3** | galanin receptor 3 | -1.16993 | 0.302744 |
| **ISL1** | ISL LIM homeobox 1 | 1.169925 | 0.302744 |
| **ASIC4** | acid sensing ion channel subunit family member 4 | 1.152003 | 0.308712 |
| **LOC728228** | long intergenic non-protein coding RNA 1433 | 1.037397 | 0.271873 |
| **ESPNL** | espin like | -1.22309 | 0.271873 |
| **SNORD91B** | small nucleolar RNA, C/D box 91B | -1.21248 | 0.308712 |
| **CR1L** | complement C3b/C4b receptor 1 like | -2.20752 | 0.308712 |
| **GPR65** | G protein-coupled receptor 65 | -2.20752 | 0.308712 |
| **HYPK** | huntingtin interacting protein K | -2.20752 | 0.308712 |
| **TRPV6** | transient receptor potential cation channel subfamily V member 6 | -2.20752 | 0.308712 |
| **MGC12916** | uncharacterized protein MGC12916 | 1.89793 | 0.308712 |
| **CNBD2** | cyclic nucleotide binding domain containing 2 | -1.18603 | 0.300631 |
| **LMOD3** | leiomodin 3 | -1.69616 | 0.308712 |
| **C22orf15** | chromosome 22 open reading frame 15 | -1.8968 | 0.302744 |
| **TPH1** | tryptophan hydroxylase 1 | 2.114409 | 0.308816 |
| **LRRC34** | leucine rich repeat containing 34 | 2.114409 | 0.308816 |
| **MIR3650** | microRNA 3650 | -1.884 | 0.302744 |
| **CD300C** | CD300c molecule | -1.30827 | 0.308712 |
| **LOC100652791** | uncharacterized LOC100652791 | -1.30827 | 0.308712 |
| **FLJ39639** | None | -1.7255 | 0.308816 |
| **CXCR5** | C-X-C motif chemokine receptor 5 | -1.31871 | 0.319069 |
| **TMEM130** | transmembrane protein 130 | 1.118762 | 0.288342 |
| **MMP1** | matrix metallopeptidase 1 | 1.284918 | 0.290463 |
| **NPY5R** | neuropeptide Y receptor Y5 | 1.585751 | 0.302744 |
| **KCNG2** | potassium voltage-gated channel modifier subfamily G member 2 | -1.25163 | 0.292099 |
| **IGLON5** | IgLON family member 5 | -1.13879 | 0.298021 |
| **LOC338651** | KRTAP5-1/KRTAP5-2 antisense RNA 1 | -1.66892 | 0.319069 |
| **RIMS2** | regulating synaptic membrane exocytosis 2 | 2.414333 | 0.319069 |
| **MIR10B** | microRNA 10b | -2 | 0.319722 |
| **LOC284933** | uncharacterized LOC284933 | -2 | 0.319722 |
| **FGG** | fibrinogen gamma chain | 1.020967 | 0.300482 |
| **P2RX3** | purinergic receptor P2X 3 | -1.33977 | 0.308712 |
| **DCST2** | DC-STAMP domain containing 2 | -1.04066 | 0.300482 |
| **ANKRD7** | ankyrin repeat domain 7 | -1.52155 | 0.308712 |
| **TENM4** | teneurin transmembrane protein 4 | 2.402033 | 0.327833 |
| **C15orf27** | transmembrane protein 266 | -1.02725 | 0.302744 |
| **ISM2** | isthmin 2 | 1.311457 | 0.302744 |
| **LOC152742** | long intergenic non-protein coding RNA 1085 | 1.311457 | 0.302744 |
| **IFI16** | interferon gamma inducible protein 16 | 1.156203 | 0.302744 |
| **TMEM88** | transmembrane protein 88 | -1.32091 | 0.308712 |
| **VPREB3** | V-set pre-B cell surrogate light chain 3 | 1.496268 | 0.308712 |
| **TMEM88B** | transmembrane protein 88B | -1.79248 | 0.343498 |
| **PODN** | podocan | -1.79248 | 0.343498 |
| **BCL6B** | BCL6B transcription repressor | -1.79248 | 0.343498 |
| **FAM26F** | calcium homeostasis modulator family member 6 | -1.79248 | 0.343498 |
| **SPATA31E1** | SPATA31 subfamily E member 1 | -1.79248 | 0.343498 |
| **SCGB1A1** | secretoglobin family 1A member 1 | -1.79248 | 0.343498 |
| **MINOS1P1** | MICOS10 pseudogene 1 | -1.79248 | 0.343498 |
| **MIR365A** | microRNA 365a | -1.79248 | 0.343498 |
| **BEST2** | bestrophin 2 | -1.79248 | 0.343498 |
| **NT5C1B** | 5'-nucleotidase, cytosolic IB | -1.79248 | 0.343498 |
| **CHDC2** | cilia and flagella associated protein 47 | -1.79248 | 0.343498 |
| **ZNF701** | zinc finger protein 701 | -1.79248 | 0.343498 |
| **LOC100505839** | SH3PXD2A antisense RNA 1 | -2.35137 | 0.344204 |
| **C19orf18** | chromosome 19 open reading frame 18 | -2.35137 | 0.344204 |
| **SLC16A8** | solute carrier family 16 member 8 | -1.1478 | 0.302744 |
| **DGCR9** | DiGeorge syndrome critical region gene 5 | -1.00194 | 0.302744 |
| **LOC100507557** | FBXO30 divergent transcript | -2.25771 | 0.302744 |
| **SERPING1** | serpin family G member 1 | 1.748026 | 0.308816 |
| **ZBTB8B** | zinc finger and BTB domain containing 8B | -2.076 | 0.335234 |
| **C1QTNF9B-AS1** | Pro-X-Gly collagen triple helix like repeat containing | -2.11748 | 0.308816 |
| **PCDHGB7** | protocadherin gamma subfamily B, 7 | -2.11748 | 0.308816 |
| **IGBP1P1** | immunoglobulin (CD79A) binding protein 1 pseudogene 1 | -1.52075 | 0.338364 |
| **FLJ44511** | None | -1.20712 | 0.308712 |
| **MIR339** | microRNA 339 | -1.13374 | 0.343498 |
| **KLHL10** | kelch like family member 10 | -2.23697 | 0.343498 |
| **USP44** | ubiquitin specific peptidase 44 | 1.152043 | 0.308712 |
| **KLHL7-AS1** | KLHL7 divergent transcript | 1.161286 | 0.308712 |
| **KRT2** | keratin 2 | -1.96142 | 0.348332 |
| **UBA7** | ubiquitin like modifier activating enzyme 7 | 1.53541 | 0.308816 |
| **LOC100286922** | DnaJ heat shock protein family (Hsp40) member B3 pseudogene | 1.347625 | 0.319069 |
| **ZNF154** | zinc finger protein 154 | -1.384 | 0.319069 |
| **DHX58** | DExH-box helicase 58 | 1.12751 | 0.339044 |
| **SPSB4** | splA/ryanodine receptor domain and SOCS box containing 4 | -1.01579 | 0.339044 |
| **SMC1B** | structural maintenance of chromosomes 1B | 1.114409 | 0.339044 |
| **CHGA** | chromogranin A | -1.09895 | 0.319069 |
| **LGI2** | leucine rich repeat LGI family member 2 | -1.15942 | 0.319069 |
| **BST2** | bone marrow stromal cell antigen 2 | 3.142125 | 0.319069 |
| **IL9R** | interleukin 9 receptor | -1.18351 | 0.352612 |
| **CCL24** | C-C motif chemokine ligand 24 | 2.183582 | 0.355561 |
| **RPL21P44** | ribosomal protein L21 pseudogene 44 | -2.36848 | 0.370216 |
| **LOC340073** | C5orf66 antisense RNA 2 | -2.36848 | 0.370216 |
| **IL1A** | interleukin 1 alpha | 1.401039 | 0.320302 |
| **CPNE5** | copine 5 | 1.004281 | 0.370216 |
| **PPP1R1C** | protein phosphatase 1 regulatory inhibitor subunit 1C | 1.780511 | 0.322685 |
| **LOC100134259** | long intergenic non-protein coding RNA 1119 | 1.187443 | 0.322876 |
| **IZUMO1** | izumo sperm-egg fusion 1 | -3.13835 | 0.358263 |
| **MX2** | MX dynamin like GTPase 2 | 2.522969 | 0.326893 |
| **FAM157A** | family with sequence similarity 157 member A | -1.21248 | 0.326893 |
| **KCNJ6** | potassium inwardly rectifying channel subfamily J member 6 | 1.034837 | 0.327097 |
| **GNGT2** | G protein subunit gamma transducin 2 | -1.29248 | 0.344497 |
| **MKNK1-AS1** | MKNK1 antisense RNA 1 | -1.79744 | 0.330462 |
| **HCRTR1** | hypocretin receptor 1 | -1.94336 | 0.362589 |
| **DLK1** | delta like non-canonical Notch ligand 1 | -1.94336 | 0.362589 |
| **MYBPHL** | myosin binding protein H like | -1.37744 | 0.370216 |
| **HIST3H3** | H3.4 histone | -1.37744 | 0.370216 |
| **PATE4** | prostate and testis expressed 4 | -1.37744 | 0.370216 |
| **LOC338817** | long intergenic non-protein coding RNA 1252 | -1.37744 | 0.370216 |
| **NELL2** | neural EGFL like 2 | -1.37744 | 0.370216 |
| **ATP12A** | ATPase H+/K+ transporting non-gastric alpha2 subunit | -1.37744 | 0.370216 |
| **SPERT** | chibby family member 2 | -1.37744 | 0.370216 |
| **PCDH20** | protocadherin 20 | -1.37744 | 0.370216 |
| **CHP2** | calcineurin like EF-hand protein 2 | -1.37744 | 0.370216 |
| **CETP** | cholesteryl ester transfer protein | -1.37744 | 0.370216 |
| **KRT40** | keratin 40 | -1.37744 | 0.370216 |
| **MIR3619** | microRNA 3619 | -1.37744 | 0.370216 |
| **MYRIP** | myosin VIIA and Rab interacting protein | -1.37744 | 0.370216 |
| **SHISA3** | shisa family member 3 | -1.37744 | 0.370216 |
| **PCDH10** | protocadherin 10 | -1.37744 | 0.370216 |
| **TMEM232** | transmembrane protein 232 | -1.37744 | 0.370216 |
| **SNCAIP** | synuclein alpha interacting protein | -1.37744 | 0.370216 |
| **C8orf34** | chromosome 8 open reading frame 34 | -1.37744 | 0.370216 |
| **ODF1** | outer dense fiber of sperm tails 1 | -1.37744 | 0.370216 |
| **SLA** | Src like adaptor | -1.37744 | 0.370216 |
| **GPC4** | glypican 4 | -1.37744 | 0.370216 |
| **FAM180B** | family with sequence similarity 180 member B | -1.37744 | 0.370216 |
| **RTN1** | reticulon 1 | -1.37744 | 0.370216 |
| **KLF15** | Kruppel like factor 15 | -1.37744 | 0.370216 |
| **ADAMTSL1** | ADAMTS like 1 | -1.37744 | 0.370216 |
| **TNFSF8** | TNF superfamily member 8 | -1.37744 | 0.370216 |
| **GJB1** | gap junction protein beta 1 | -1.37744 | 0.370216 |
| **FXYD3** | FXYD domain containing ion transport regulator 3 | -3.54432 | 0.343498 |
| **NRN1L** | neuritin 1 like | -2.08264 | 0.335234 |
| **MAPK4** | mitogen-activated protein kinase 4 | 1.705929 | 0.348332 |
| **BTN1A1** | butyrophilin subfamily 1 member A1 | -3.11548 | 0.352453 |
| **ANKRD31** | ankyrin repeat domain 31 | 1.36983 | 0.339044 |
| **ANP32AP1** | acidic nuclear phosphoprotein 32 family member A pseudogene 1 | -1.90368 | 0.371843 |
| **GPR156** | G protein-coupled receptor 156 | 1.07241 | 0.340036 |
| **CHST5** | carbohydrate sulfotransferase 5 | -1.31221 | 0.340549 |
| **C12orf50** | chromosome 12 open reading frame 50 | -2.64386 | 0.362589 |
| **LOC100507584** | long intergenic non-protein coding RNA 1016 | -2.64386 | 0.362589 |
| **ELOVL3** | ELOVL fatty acid elongase 3 | 1.433631 | 0.352515 |
| **C3P1** | complement component 3 precursor pseudogene | -1.5897 | 0.352612 |
| **ACOXL** | acyl-CoA oxidase like | -1.3208 | 0.352612 |
| **FRG2B** | FSHD region gene 2 family member B | -2.41504 | 0.370216 |
| **CCDC67** | deuterosome assembly protein 1 | -2.41504 | 0.370216 |
| **BCL2L14** | BCL2 like 14 | -2.41504 | 0.370216 |
| **FUT3** | fucosyltransferase 3 (Lewis blood group) | -2.41504 | 0.370216 |
| **SNORA70F** | small nucleolar RNA, H/ACA box 70F | -2.41504 | 0.370216 |
| **SPATC1L** | spermatogenesis and centriole associated 1 like | -2.41504 | 0.370216 |
| **CDC20B** | cell division cycle 20B | -2.41504 | 0.370216 |
| **SLC44A4** | solute carrier family 44 member 4 | -2.41504 | 0.370216 |
| **PAPOLB** | poly(A) polymerase beta | -2.41504 | 0.370216 |
| **SH3GL2** | SH3 domain containing GRB2 like 2, endophilin A1 | -2.41504 | 0.370216 |
| **PAEP** | progestagen associated endometrial protein | -2.41504 | 0.370216 |
| **LOC646214** | p21 (RAC1) activated kinase 2 pseudogene | -1.55601 | 0.343498 |
| **PI3** | peptidase inhibitor 3 | 1.005053 | 0.343498 |
| **APOL3** | apolipoprotein L3 | 2.321928 | 0.370216 |
| **SEMA3D** | semaphorin 3D | 2.321928 | 0.370216 |
| **VWA3A** | von Willebrand factor A domain containing 3A | -1.70752 | 0.371843 |
| **AARD** | alanine and arginine rich domain containing protein | -1.70752 | 0.371843 |
| **FLJ31813** | family with sequence similarity 21, member A pseudogene | -1.432 | 0.352612 |
| **DNAH2** | dynein axonemal heavy chain 2 | -1.432 | 0.352612 |
| **UBOX5-AS1** | UBOX5 antisense RNA 1 | -1.432 | 0.352612 |
| **COL4A2-AS1** | COL4A2 antisense RNA 1 | -1.1912 | 0.352612 |
| **GPR1** | G protein-coupled receptor 1 | -2.22239 | 0.371843 |
| **LY9** | lymphocyte antigen 9 | -1.60814 | 0.343498 |
| **PPP1R1A** | protein phosphatase 1 regulatory inhibitor subunit 1A | 1.382402 | 0.370216 |
| **C4orf26** | odontogenesis associated phosphoprotein | 1.382402 | 0.370216 |
| **FBXO24** | F-box protein 24 | 1.211531 | 0.343498 |
| **MILR1** | mast cell immunoglobulin like receptor 1 | 1.067681 | 0.343498 |
| **GSDMC** | gasdermin C | -2.10731 | 0.370216 |
| **RORC** | RAR related orphan receptor C | -2.05889 | 0.371843 |
| **FLJ42875** | None | -2 | 0.371843 |
| **FLG2** | filaggrin family member 2 | -2 | 0.371843 |
| **POU5F1P3** | POU class 5 homeobox 1 pseudogene 3 | -2 | 0.371843 |
| **POSTN** | periostin | -2 | 0.371843 |
| **SERPINE3** | serpin family E member 3 | -2 | 0.371843 |
| **BTBD17** | BTB domain containing 17 | -2 | 0.371843 |
| **UTS2R** | urotensin 2 receptor | -2 | 0.371843 |
| **ELAVL3** | ELAV like RNA binding protein 3 | -2 | 0.371843 |
| **SIGLEC14** | sialic acid binding Ig like lectin 14 | -2 | 0.371843 |
| **GNLY** | granulysin | -2 | 0.371843 |
| **MIR125B2** | microRNA 125b-2 | -2 | 0.371843 |
| **NCAM2** | neural cell adhesion molecule 2 | -2 | 0.371843 |
| **UPB1** | beta-ureidopropionase 1 | -2 | 0.371843 |
| **GP5** | glycoprotein V platelet | -2 | 0.371843 |
| **METTL24** | methyltransferase like 24 | -2 | 0.371843 |
| **GCK** | glucokinase | -2 | 0.371843 |
| **UG0898H09** | sodium/potassium transporting ATPase interacting 3 | -2 | 0.371843 |
| **TTTY14** | testis-specific transcript, Y-linked 14 | -2 | 0.371843 |
| **SYCE3** | synaptonemal complex central element protein 3 | -1.95936 | 0.374509 |
| **C1orf51** | circadian associated repressor of transcription | 1.638958 | 0.34665 |
| **FLJ46300** | None | -1.52832 | 0.358263 |
| **AVPR2** | arginine vasopressin receptor 2 | 1.917538 | 0.378259 |
| **WTH3DI** | RAB6D, member RAS oncogene family | 1.906891 | 0.378259 |
| **ARG1** | arginase 1 | 1.906891 | 0.378259 |
| **MRGPRF** | MAS related GPR family member F | -1.70752 | 0.370216 |
| **LTA** | lymphotoxin alpha | -1.70752 | 0.370216 |
| **LAMP3** | lysosomal associated membrane protein 3 | 1.776148 | 0.348332 |
| **HIST1H2BL** | H2B clustered histone 13 | -1.15365 | 0.348332 |
| **RIIAD1** | regulatory subunit of type II PKA R-subunit domain containing 1 | -1.6112 | 0.370216 |
| **LINC00612** | long intergenic non-protein coding RNA 612 | -2.11595 | 0.359836 |
| **TNFRSF4** | TNF receptor superfamily member 4 | -1.80735 | 0.379265 |
| **MIR4717** | microRNA 4717 | 1.807355 | 0.379265 |
| **USHBP1** | USH1 protein network component harmonin binding protein 1 | 1.807355 | 0.379265 |
| **CHADL** | chondroadherin like | 1.807355 | 0.379265 |
| **SPARCL1** | SPARC like 1 | 1.807355 | 0.379265 |
| **UNC93A** | unc-93 homolog A | 1.807355 | 0.379265 |
| **MIR589** | microRNA 589 | 1.807355 | 0.379265 |
| **NSUN5P2** | NSUN5 pseudogene 2 | 1.807355 | 0.379265 |
| **PMS2L2** | PMS1 homolog 2, mismatch repair system component pseudogene 2 | 1.807355 | 0.379265 |
| **IL33** | interleukin 33 | 1.807355 | 0.379265 |
| **LPAR4** | lysophosphatidic acid receptor 4 | -1.80735 | 0.379265 |
| **SCARNA9** | small Cajal body-specific RNA 9 | 1.703787 | 0.370216 |
| **APOBR** | apolipoprotein B receptor | -1.87118 | 0.348691 |
| **KRT6B** | keratin 6B | 1.953445 | 0.379265 |
| **GABRR2** | gamma-aminobutyric acid type A receptor subunit rho2 | 1.953445 | 0.379265 |
| **C10orf62** | chromosome 10 open reading frame 62 | -1.5 | 0.36192 |
| **IL7R** | interleukin 7 receptor | 2.516916 | 0.371843 |
| **NLRP12** | NLR family pyrin domain containing 12 | -1.20752 | 0.379265 |
| **KCNT1** | potassium sodium-activated channel subfamily T member 1 | -1.20752 | 0.379265 |
| **SRGAP2C** | SLIT-ROBO Rho GTPase activating protein 2C | -2.55889 | 0.379265 |
| **CCL7** | C-C motif chemokine ligand 7 | -2.55889 | 0.379265 |
| **GSTT2** | glutathione S-transferase theta 2 (gene/pseudogene) | -2.55889 | 0.379265 |
| **ESPNP** | espin pseudogene | -1.58496 | 0.390537 |
| **ZFP69** | ZFP69 zinc finger protein | 1.584963 | 0.390537 |
| **LURAP1** | leucine rich adaptor protein 1 | -1.58496 | 0.390537 |
| **LOC729987** | long intergenic non-protein coding RNA 1776 | -1.58496 | 0.390537 |
| **NBPF24** | NBPF member 11 | -1.58496 | 0.390537 |
| **SLAMF9** | SLAM family member 9 | -1.58496 | 0.390537 |
| **LOC100505795** | PBX1 antisense RNA 1 | -1.58496 | 0.390537 |
| **GPR25** | G protein-coupled receptor 25 | -1.58496 | 0.390537 |
| **OR51B5** | olfactory receptor family 51 subfamily B member 5 | -1.58496 | 0.390537 |
| **FOLR4** | IZUMO1 receptor, JUNO | -1.58496 | 0.390537 |
| **C11orf44** | long intergenic non-protein coding RNA 2873 | -1.58496 | 0.390537 |
| **LOC100128239** | long intergenic non-protein coding RNA 2731 | -1.58496 | 0.390537 |
| **CACNA1C-AS1** | CACNA1C antisense RNA 1 | -1.58496 | 0.390537 |
| **GOLGA8I** | golgin A8 family member I, pseudogene | -1.58496 | 0.390537 |
| **DUOXA2** | dual oxidase maturation factor 2 | -1.58496 | 0.390537 |
| **C1QTNF8** | C1q and TNF related 8 | -1.58496 | 0.390537 |
| **EIF3C** | eukaryotic translation initiation factor 3 subunit C | -1.58496 | 0.390537 |
| **KRT27** | keratin 27 | -1.58496 | 0.390537 |
| **KRT42P** | keratin 42 pseudogene | -1.58496 | 0.390537 |
| **DLX3** | distal-less homeobox 3 | -1.58496 | 0.390537 |
| **MIR1539** | microRNA 1539 | -1.58496 | 0.390537 |
| **MC4R** | melanocortin 4 receptor | -1.58496 | 0.390537 |
| **VSTM2B** | V-set and transmembrane domain containing 2B | -1.58496 | 0.390537 |
| **PTGIR** | prostaglandin I2 receptor | 1.584963 | 0.390537 |
| **ZNF808** | zinc finger protein 808 | -1.58496 | 0.390537 |
| **ZNF600** | zinc finger protein 600 | 1.584963 | 0.390537 |
| **NXPH2** | neurexophilin 2 | -1.58496 | 0.390537 |
| **LY75** | lymphocyte antigen 75 | -1.58496 | 0.390537 |
| **LOC100130451** | SPAG16 divergent transcript | -1.58496 | 0.390537 |
| **MOGAT1** | monoacylglycerol O-acyltransferase 1 | -1.58496 | 0.390537 |
| **FLJ43879** | None | -1.58496 | 0.390537 |
| **CCBP2** | atypical chemokine receptor 2 | -1.58496 | 0.390537 |
| **PROM1** | prominin 1 | 1.584963 | 0.390537 |
| **UGT2B4** | UDP glucuronosyltransferase family 2 member B4 | -1.58496 | 0.390537 |
| **MIR302A** | microRNA 302a | -1.58496 | 0.390537 |
| **PKD2L2** | polycystin 2 like 2, transient receptor potential cation channel | -1.58496 | 0.390537 |
| **ADCYAP1R1** | ADCYAP receptor type I | -1.58496 | 0.390537 |
| **MIR5090** | microRNA 5090 | -1.58496 | 0.390537 |
| **FOXP2** | forkhead box P2 | -1.58496 | 0.390537 |
| **WNT16** | Wnt family member 16 | -1.58496 | 0.390537 |
| **FABP5P3** | fatty acid binding protein 5 pseudogene 3 | -1.58496 | 0.390537 |
| **MAL2** | mal, T cell differentiation protein 2 (gene/pseudogene) | -1.58496 | 0.390537 |
| **KLHL38** | kelch like family member 38 | -1.58496 | 0.390537 |
| **MIR661** | microRNA 661 | -1.58496 | 0.390537 |
| **LOC340508** | growth arrest specific 2 like 1 pseudogene 2 | -1.58496 | 0.390537 |
| **FUT7** | fucosyltransferase 7 | -1.58496 | 0.390537 |
| **BTK** | Bruton tyrosine kinase | -1.58496 | 0.390537 |
| **AKAP14** | A-kinase anchoring protein 14 | -1.58496 | 0.390537 |
| **SPANXA2-OT1** | SPANXA2 overlapping transcript 1 | -1.58496 | 0.390537 |
| **LINC00475** | long intergenic non-protein coding RNA 475 | -1.86848 | 0.371843 |
| **CLIC2** | chloride intracellular channel 2 | -1.82238 | 0.352612 |
| **LOC643339** | uncharacterized LOC643339 | -2.68267 | 0.371843 |
| **C4BPA** | complement component 4 binding protein alpha | 1.275342 | 0.352612 |
| **OR10AD1** | olfactory receptor family 10 subfamily AD member 1 | -1.47393 | 0.400022 |
| **MIR3682** | microRNA 3682 | 1.473931 | 0.400022 |
| **HIST1H4B** | H4 clustered histone 2 | -1.51487 | 0.390537 |
| **GAS1** | growth arrest specific 1 | -1.51487 | 0.390537 |
| **PRELP** | proline and arginine rich end leucine rich repeat protein | -1.41504 | 0.406485 |
| **SLC6A20** | solute carrier family 6 member 20 | -1.41504 | 0.406485 |
| **TREML3P** | triggering receptor expressed on myeloid cells like 3, pseudogene | 1.427095 | 0.370216 |
| **CA7** | carbonic anhydrase 7 | 1.249631 | 0.358263 |
| **SH3GL3** | SH3 domain containing GRB2 like 3, endophilin A3 | -1.08496 | 0.371843 |
| **TSPAN16** | tetraspanin 16 | -1.08496 | 0.371843 |
| **APOC1P1** | apolipoprotein C1 pseudogene 1 | -1.08496 | 0.371843 |
| **EGFR-AS1** | EGFR antisense RNA 1 | -1.17323 | 0.359205 |
| **CARD16** | caspase recruitment domain family member 16 | -2 | 0.390537 |
| **CTAGE1** | cutaneous T cell lymphoma-associated antigen 1 | -2 | 0.390537 |
| **GPRASP2** | G protein-coupled receptor associated sorting protein 2 | 1.179291 | 0.359836 |
| **UGT2A3** | UDP glucuronosyltransferase family 2 member A3 | -1.22331 | 0.370216 |
| **POU3F3** | POU class 3 homeobox 3 | -1.97045 | 0.360303 |
| **VTRNA1-2** | vault RNA 1-2 | -1.44448 | 0.390537 |
| **GUCA2B** | guanylate cyclase activator 2B | -2.55774 | 0.393941 |
| **SMCR5** | Smith-Magenis syndrome chromosome region, candidate 5 | -1.86875 | 0.370216 |
| **NAGPA-AS1** | NAGPA antisense RNA 1 | -2.89022 | 0.379265 |
| **SLC24A4** | solute carrier family 24 member 4 | -1.06479 | 0.362589 |
| **CDRT15** | CMT1A duplicated region transcript 15 | -1.18288 | 0.362589 |
| **STON1** | stonin 1 | 1.720675 | 0.363339 |
| **LOC728175** | long intergenic non-protein coding RNA 2363 | 1.48864 | 0.400022 |
| **PCDHGC5** | protocadherin gamma subfamily C, 5 | 1.48864 | 0.400022 |
| **SFTPC** | surfactant protein C | 1.48864 | 0.400022 |
| **CXCL12** | C-X-C motif chemokine ligand 12 | -2.11441 | 0.379265 |
| **NKX2-3** | NK2 homeobox 3 | -2.11441 | 0.379265 |
| **CNGA1** | cyclic nucleotide gated channel subunit alpha 1 | -2.11441 | 0.379265 |
| **KRT222** | keratin 222 | 1.256562 | 0.370216 |
| **NME8** | NME/NM23 family member 8 | -1.85025 | 0.371843 |
| **PRAP1** | proline rich acidic protein 1 | -1.76922 | 0.371843 |
| **CBWD3** | COBW domain containing 3 | 1.007456 | 0.371843 |
| **C1orf65** | coiled-coil domain containing 185 | -1.43231 | 0.371843 |
| **NOS2** | nitric oxide synthase 2 | 1.817113 | 0.370216 |
| **PLAC4** | placenta enriched 4 | -1.19616 | 0.380844 |
| **STAB1** | stabilin 1 | -1.19616 | 0.380844 |
| **ST7-OT4** | ST7 overlapping transcript 4 | -1.01487 | 0.407401 |
| **PRSS56** | serine protease 56 | 1.076629 | 0.371843 |
| **NODAL** | nodal growth differentiation factor | -2.42985 | 0.371843 |
| **MYO15A** | myosin XVA | 1.255483 | 0.370216 |
| **MIR1469** | microRNA 1469 | -1.52832 | 0.390537 |
| **MCF2** | MCF.2 cell line derived transforming sequence | -1.52832 | 0.390537 |
| **TMEM255B** | transmembrane protein 255B | 1.223401 | 0.370216 |
| **C2orf57** | testis expressed 44 | -1.29248 | 0.410082 |
| **MIR146A** | microRNA 146a | -1.29248 | 0.410082 |
| **MLN** | motilin | -1.29248 | 0.410082 |
| **SLITRK4** | SLIT and NTRK like family member 4 | -1.29248 | 0.410082 |
| **MIR452** | microRNA 452 | -1.29248 | 0.410082 |
| **SNORD12B** | small nucleolar RNA, C/D box 12B | 1.508536 | 0.370216 |
| **SNORA2B** | small nucleolar RNA, H/ACA box 2B | -1.78875 | 0.371843 |
| **OSM** | oncostatin M | -1.73697 | 0.390537 |
| **SPINK1** | serine peptidase inhibitor Kazal type 1 | -1.86314 | 0.370216 |
| **PROSER2-AS1** | PROSER2 antisense RNA 1 | -1.37446 | 0.370216 |
| **VSTM1** | V-set and transmembrane domain containing 1 | 1.76648 | 0.373605 |
| **HAPLN2** | hyaluronan and proteoglycan link protein 2 | -1.77398 | 0.370216 |
| **TDRD12** | tudor domain containing 12 | -2.21462 | 0.390537 |
| **PSTPIP1** | proline-serine-threonine phosphatase interacting protein 1 | -1.58123 | 0.374509 |
| **DUSP26** | dual specificity phosphatase 26 | -1.20192 | 0.374509 |
| **CREB3L1** | cAMP responsive element binding protein 3 like 1 | 1.352965 | 0.370216 |
| **LOC339535** | long intergenic non-protein coding RNA 1139 | -1.576 | 0.390537 |
| **CALCB** | calcitonin related polypeptide beta | -2.6112 | 0.390537 |
| **CPSF4L** | cleavage and polyadenylation specific factor 4 like | -1.80728 | 0.376807 |
| **MEIS1-AS3** | MEIS1 antisense RNA 3 | -1.80728 | 0.376807 |
| **MIR612** | microRNA 612 | -1.69227 | 0.393941 |
| **ALDOB** | aldolase, fructose-bisphosphate B | -1.69227 | 0.393941 |
| **LOC100507410** | C1QTNF1 antisense RNA 1 | -1.25163 | 0.371843 |
| **DPCR1** | mucin like 3 | 1.483693 | 0.371843 |
| **RNF5P1** | ring finger protein 5 pseudogene 1 | -1.50501 | 0.379265 |
| **C4orf19** | chromosome 4 open reading frame 19 | -1.21248 | 0.379265 |
| **MYO3A** | myosin IIIA | -1.29248 | 0.392625 |
| **MEIG1** | meiosis/spermiogenesis associated 1 | 1.357886 | 0.371843 |
| **LINC00525** | long intergenic non-protein coding RNA 525 | 1.067463 | 0.371843 |
| **SH2D3C** | SH2 domain containing 3C | 1.042249 | 0.379265 |
| **TMEM255A** | transmembrane protein 255A | 1.608536 | 0.371843 |
| **HIST1H2BN** | H2B clustered histone 15 | -1.14554 | 0.371843 |
| **OR2B6** | olfactory receptor family 2 subfamily B member 6 | 1.150404 | 0.406485 |
| **NUP210L** | nucleoporin 210 like | 1.055681 | 0.399889 |
| **C17orf61-PLSCR3** | TMEM256-PLSCR3 readthrough (NMD candidate) | -2.19724 | 0.406765 |
| **RAG2** | recombination activating 2 | 1.529447 | 0.399889 |
| **LOC151009** | long intergenic non-protein coding RNA 1106 | -1.01487 | 0.400022 |
| **LOC100507299** | SMC5 antisense RNA 1 (head to head) | 1.592888 | 0.386505 |
| **TTYH1** | tweety family member 1 | -1.57011 | 0.371843 |
| **LOC100506776** | T cell receptor gamma locus antisense RNA 1 | -1.5889 | 0.374509 |
| **TBC1D3P1-DHX40P1** | TBC1D3P1-DHX40P1 readthrough, transcribed pseudogene | -1.25097 | 0.374509 |
| **P2RX2** | purinergic receptor P2X 2 | -1.73348 | 0.390537 |
| **DACT3-AS1** | DACT3 antisense RNA 1 | -1.73348 | 0.390537 |
| **UGT2B7** | UDP glucuronosyltransferase family 2 member B7 | 1.323757 | 0.376493 |
| **SNORA7B** | small nucleolar RNA, H/ACA box 7B | 1.260618 | 0.407401 |
| **DPRXP4** | divergent-paired related homeobox pseudogene 4 | -1.09376 | 0.378259 |
| **GATA3-AS1** | GATA3 antisense RNA 1 | -1.8912 | 0.378259 |
| **ASB18** | ankyrin repeat and SOCS box containing 18 | -1.70593 | 0.378736 |
| **MMP19** | matrix metallopeptidase 19 | -1.29248 | 0.407401 |
| **MTRNR2L4** | MT-RNR2 like 4 | -1.29248 | 0.407401 |
| **RFPL3S** | RFPL3 antisense | -1.29248 | 0.407401 |
| **IFNA1** | interferon alpha 1 | -1.29248 | 0.407401 |
| **BTBD18** | BTB domain containing 18 | -1.40368 | 0.407785 |
| **CST4** | cystatin S | -1.40368 | 0.407785 |
| **SUN3** | Sad1 and UNC84 domain containing 3 | -1.40368 | 0.407785 |
| **CATSPER2P1** | cation channel sperm associated 2 pseudogene 1 | 1.081883 | 0.379265 |
| **MATN1** | matrilin 1 | -1.68506 | 0.390537 |
| **SNORD38A** | small nucleolar RNA, C/D box 38A | -2.18144 | 0.379265 |
| **LOC256880** | H2AZ1 divergent transcript | -1.08496 | 0.409857 |
| **TCTE3** | t-complex-associated-testis-expressed 3 | -1.28389 | 0.379265 |
| **ADHFE1** | alcohol dehydrogenase iron containing 1 | -1.04596 | 0.379265 |
| **PPP1R27** | protein phosphatase 1 regulatory subunit 27 | -1.45706 | 0.390537 |
| **STXBP5-AS1** | STXBP5 antisense RNA 1 | -1.13947 | 0.390537 |
| **LINC00607** | long intergenic non-protein coding RNA 607 | 1.139828 | 0.390537 |
| **SNORD76** | small nucleolar RNA, C/D box 76 | 1.080941 | 0.379265 |
| **MIR23A** | microRNA 23a | -1 | 0.380964 |
| **FLJ35282** | None | 1.495002 | 0.393941 |
| **ZNF595** | zinc finger protein 595 | 1.204275 | 0.382565 |
| **KCNT2** | potassium sodium-activated channel subfamily T member 2 | 1.5908 | 0.399889 |
| **C3orf20** | chromosome 3 open reading frame 20 | -1.76257 | 0.390537 |
| **ZNF785** | zinc finger protein 785 | -1.00931 | 0.390537 |
| **SAMD10** | sterile alpha motif domain containing 10 | -1.57318 | 0.390537 |
| **SNORD33** | small nucleolar RNA, C/D box 33 | 1.199307 | 0.390537 |
| **LEFTY1** | left-right determination factor 1 | -1 | 0.390537 |
| **LRRN3** | leucine rich repeat neuronal 3 | -1 | 0.390537 |
| **PTGER2** | prostaglandin E receptor 2 | 1.162466 | 0.390537 |
| **SNORD45B** | small nucleolar RNA, C/D box 45B | -1.16863 | 0.390537 |
| **GDF6** | growth differentiation factor 6 | -2.0959 | 0.390537 |
| **CRABP1** | cellular retinoic acid binding protein 1 | 1.106204 | 0.390537 |
| **KEL** | Kell metallo-endopeptidase (Kell blood group) | -1.50683 | 0.406765 |
| **SNORA57** | small nucleolar RNA, H/ACA box 57 | 1.061886 | 0.390537 |
| **PLCB2** | phospholipase C beta 2 | -1.25323 | 0.390537 |
| **FAM19A3** | TAFA chemokine like family member 3 | -1.95158 | 0.40895 |
| **LOC101059948** | uncharacterized LOC101059948 | -1.03952 | 0.393756 |
| **FAM133B** | family with sequence similarity 133 member B | -1.53059 | 0.410082 |
| **SRMS** | src-related kinase lacking C-terminal regulatory tyrosine and N-terminal myristylation sites | -2.60319 | 0.410082 |
| **PIWIL2** | piwi like RNA-mediated gene silencing 2 | 1.219933 | 0.393941 |
| **PF4** | platelet factor 4 | 1.017314 | 0.394382 |
| **LOC100507472** | uncharacterized LOC100507472 | -1.73834 | 0.397785 |
| **TMEM26** | transmembrane protein 26 | 1.422493 | 0.399283 |
| **FLJ44313** | None | 1.100462 | 0.399889 |
| **SHANK1** | SH3 and multiple ankyrin repeat domains 1 | -1.76043 | 0.399889 |
| **PCDHB16** | protocadherin beta 16 | -1.15721 | 0.403489 |
| **CYP27A1** | cytochrome P450 family 27 subfamily A member 1 | -1.17761 | 0.407401 |
| **SPOCK3** | SPARC (osteonectin), cwcv and kazal like domains proteoglycan 3 | -1.20599 | 0.407401 |
| **ZSCAN12P1** | zinc finger and SCAN domain containing 12 pseudogene 1 | -1.81099 | 0.407401 |
| **CILP2** | cartilage intermediate layer protein 2 | -1.0912 | 0.409592 |
| **IGF2** | insulin like growth factor 2 | 1.093762 | 0.410082 |
| **RASSF9** | Ras association domain family member 9 | 1.094447 | 0.410082 |
